# A systematic review of non-contact medical IoT systems for physiological biomarker monitoring

**DOI:** 10.3389/fmedt.2026.1917332

**Published:** 2026-10-15

**Authors:** Bithi Banik, Saptanil Ghose, Ahnaf Hasnain Nahiun, Yuan Lin, Andrii Shalaginov, Debasish Ghose

**Affiliations:** 1 Department of Technology, Kristiania University of Applied Sciences, Bergen, Norway; 2 Department of Computer Science, American International University, Dhaka, Bangladesh

**Keywords:** federated learning, medical IoT, non-contact vital sign monitoring, physiological biomarkers, PRISMA

## Abstract

**Introduction:**

Non-contact medical Internet of Things systems measure physiological biomarkers such as heart rate (HR), respiration rate (RR), temperature, oxygen saturation (SpO₂), and blood pressure (BP) without physical contact with the patient. These systems combine sensing modalities including cameras, radar, Wi-Fi, acoustic, and infrared sensors with signal processing and learning algorithms to deliver comfortable, non-invasive monitoring. Prior reviews have largely examined individual sensing modalities or signal processing techniques in isolation, leaving cross-modality system architecture and privacy-preserving design underexamined.

**Methods:**

We conducted a systematic review following PRISMA guidelines and analyzed 88 recent studies of non-contact monitoring systems. Studies were characterized by sensing modality, system architecture, target physiological parameter, validation setting, and reported performance. We additionally assessed each study for methodological quality and examined the extent to which privacy-preserving mechanisms were incorporated into system design.

**Results:**

Radar and vision-based systems dominate the corpus, together accounting for approximately three-quarters of included studies, while sound, Wi-Fi, embedded sensor, and mixed-modality designs remain at an earlier stage of validation. Research effort is heavily concentrated on two parameters: RR appears in 83% of studies and HR in 67%, while no other parameter exceeds 11%. BP, SpO₂, and temperature therefore remain largely unaddressed by contactless methods. Heterogeneous reporting metrics across studies prevent direct statistical comparison of performance between modalities.

**Discussion:**

The findings indicate that modality selection should be driven by deployment context rather than headline accuracy. Radar suits wards and intensive care units where patients are covered or moving, vision and sound suit structured clinical spaces with stable conditions, embedded sensor designs suit eldercare beds and chairs, and mixed radar and vision systems suit precision-critical settings such as neonatal and postoperative monitoring. Persistent challenges include signal reliability in uncontrolled environments, limited benchmarking due to the absence of standardized datasets, privacy and ethical concerns, and poor integration with existing healthcare infrastructure. Building on these insights, we propose a conceptual federated, edge-based learning framework, presented as a design proposal for future implementation rather than a validated system, and outline research directions to accelerate clinical translation.

## 1 Introduction

Physiological biomarker monitoring is a crucial aspect of healthcare that involves the regular measurement and tracking of key vital signs to assess a patient’s health status. The primary vital signs typically monitored include HR, RR, BP, body temperature, and SpO${}_{2}$. These vital signs are considered the most precise predictors of a patient’s clinical worsening (1). A physiological biomarker or vital sign monitoring system is a comprehensive arrangement designed to continuously or periodically measure and record a patient’s vital signs. These systems are crucial in various healthcare environments, including hospitals, clinics, and home care, to ensure the timely detection of any changes in a patient’s health. Nurses and doctors typically use devices such as electrodes for ECG monitoring, BP cuffs, thermometers for body temperature, and pulse oximeters for SpO${}_{2}$. However, factors like the heavy workload of healthcare providers and a lack of clinical knowledge can impact the quality of continuous vital sign monitoring (2).

Traditional contact-based vital sign monitoring methods require direct physical contact, and many of these approaches are invasive. IoT devices that necessitate physical contact can cause skin irritation or damage, make patients uncomfortable, introduce risks of infection and cross-contamination, and limit mobility (3). These negative consequences can affect the accuracy and consistency of continuous data collection. As a result, non-contact vital sign monitoring is increasingly becoming a primary focus. Non-contact vital sign monitoring refers to the use of technology to measure and track a patient’s vital signs without the need for physical contact with the body. This approach leverages advanced sensors and imaging technologies to detect physiological parameters such as HR, RR, body temperature, and SpO${}_{2}$ from a distance. With advancements in healthcare technology, the integration of the IoT and telemedicine has significantly progressed the field (4). The maturation of IoT sensing, wireless networks, and AI-driven analytics has driven comparable transformations across other data-intensive domains, such as precision agriculture (5), where distributed sensors and machine learning are used to enable continuous, automated monitoring at scale. In healthcare, this convergence underpins the shift from intermittent, manual measurement toward continuous physiological monitoring.

To date, several authors have worked with non-contact vital sign monitoring. Some have used radar-based technology (3, 6–11) for data acquisition. Image-based technology is also gaining popularity as it can provide additional information that radar systems cannot, such as facial expressions (12) and skin temperature (13). Several researchers have included radar-based technology with other sensors like fingertip pulse sensors and chest belts to provide reference signals for the acquisition of vital signs to enhance the accuracy of information from a distance (14).

It is essential to understand the fundamental principles and potential opportunities along with the limitations of contactless vital sign monitoring systems before working with them. Signal distortion and nonlinearity (15), motion artifacts (7), and environmental factors (16) are a few limitations of this system. Some potential opportunities like continuous health monitoring, integration with smart systems (16), and unobtrusive monitoring (8) have captured the attention of researchers.

There are a few review works (17–21) that have been published to gather knowledge on contactless vital sign monitoring systems. For instance, the author of (17) conducted a systematic review on non-contact vision-based techniques of vital sign monitoring using the PRISMA guidelines. An overview of video-based vital sign monitoring is discussed in Molinaro et al. (20), while Singh et al. (18) has focused on IoT and sleep applications related to HR and RR. Another review paper (19) provides a comprehensive technological review on the current state-of-the-art of non-contact vital sign measurements using radar-based technology. More recently, Liu et al. (21) reviewed nanotechnology-driven non-contact sensors for remote cardiovascular monitoring within intelligent nursing, identifying clinical validation, data processing, and large-scale implementation as the principal barriers to translation; however, its scope is confined to nanomaterial-based sensing and to cardiovascular parameters (HR, RR, and BP). A qualitative comparison among the review studies is highlighted in Table 1. It is worth noting that previous reviews on non-contact vital sign monitoring have primarily focused on specific system designs, sensing materials, or clinical applications, often lacking a rigorous and comprehensive analysis that integrates multiple technologies and vital signs. In contrast, this study offers a comprehensive review of major system designs used in non-contact vital sign monitoring, including radar, imaging, acoustic, Wi-Fi, and sensor-based systems, while covering key physiological parameters including HR, RR, SpO${}_{2}$, temperature, BP, HRV, and IBI. It aims to present an integrated perspective on their respective advantages and limitations within a single, unified survey. A list of abbreviations used throughout this article is provided in Table 2 for clarity.

**Table 1:** Qualitative comparison with the existing reviews on non-contact vital sign monitoring.

Ref.	Type	System Design	Opportunities	Challenges	Vital Signs
(22)	Comprehensive Survey	Vision, Radar	No	No	HR, RR, BP, Temperature
(23)	Detail Survey	Radar	No	No	HR, RR, BP, Temperature
(17)	Systematic Review using PRISMA	Vision	No	No	HR, BP, SpO${}_{2}$
(18)	Technological Review	Radar	Yes	Yes	HR, RR
(19)	Comprehensive Survey	Radar and Vision	Yes	Yes	HR, RR
(20)	Overview	Vision	No	No	HR, RR, BP, SpO${}_{2}$
(21)	Narrative Review	Radar and Vision	Yes	Yes	HR, RR, BP
This study	Systematic Review using PRISMA	Radar, Vision, Sensor, Sound, Wi-Fi, and Mixed	Yes	Yes	HR, RR, BP, SpO${}_{2}$, Temperature, HRV, IBI

**Table 2:** List of abbreviations.

Abbreviation	Definition
ACL	Adaptive Cancellation Loop
AI	Artificial Intelligence
ANF	Adaptive Notch Filter
AoA	Angle of Arrival
BBI	Beat-to-Beat Interval
BPM	Beats Per Minute
BP	Blood Pressure
BrPM	Breaths Per Minute
BVP	Blood Volume Pulse
CCC	Concordance Correlation Coefficient
CFR	Channel Frequency Response
CIR	Channel Impulse Response
cPCA	Contrastive Principal Component Analysis
CW	Continuous Wave
DBP	Diastolic Blood Pressure
DL	Deep Learning
DP	Dynamic Programming
ECG	Electrocardiogram
EPS	Electric Potential Sensors
EVM	Eulerian Video Magnification
FFT	Fast Fourier Transform
FL	Federated Learning
FMCW	Frequency-Modulated Continuous-Wave
HR	Heart Rate
HRV	Heart Rate Variability
ICC	Intraclass Correlation Coefficient
IoT	Internet of Things
IR-UWB	Impulse-Radio Ultra-Wideband
LOS	Line-of-Sight
MAE	Mean Absolute Error
MAPE	Mean Absolute Percentage Error
ME	Mean Error
ML	Machine Learning
MMwave	Millimeter Wave
NLMS	Normalized Least Mean Square
OPOIRES	Optimal Points-Of-Interest based RR Estimation
PCA	Principal Component Analysis
PCC	Pearson Correlation Coefficients
PLI	Power Line Interference
PRISMA	Preferred Reporting Items for Systematic Reviews
	Meta-Analyses
RMSD	Root Mean Squared Difference
RMSE	Root Mean Square Error
ROI	Region of Interest
RQs	Research Questions
RR	Respiration Rate
RPM	Respirations Per Minute
SBP	Systolic Blood Pressure
SE	Standard Error
SINR	Signal-to-Interference-Plus-Noise Ratio
SNR	Signal-to-Noise Ratio
SpO${}_{2}$	Oxygen Saturation
STFT	Short-Time Fourier Transform
TIA	Transimpedance Amplifier
UWB	Ultra-Wideband
vPPG	Video Photoplethysmography
VLS	Visible Light Sensing

## 2 Review methodology

This systematic review followed the PRISMA 2020 guidelines (24). The review targeted peer-reviewed journal articles on non-contact vital sign monitoring published between January 1, 2019 and July 25, 2026. The methodological workflow comprised research question formulation, database selection, systematic searching, multi-stage screening, quality assessment, and data extraction.

### 2.1 Research question identification

A preliminary qualitative comparison (Table 1) and the analysis in Section 1 highlighted a research gap concerning the sensing techniques, opportunities, and challenges associated with non-contact vital sign monitoring. Accordingly, the following research questions (RQs) were formulated:

- **RQ1:** Which system designs and techniques are employed for non-contact vital sign monitoring?
- **RQ2:** What are the opportunities associated with non-contact vital sign monitoring?
- **RQ3:** What are the challenges associated with non-contact vital sign monitoring?

### 2.2 Database selection

Six scientific databases were selected for their broad coverage of engineering, computer science, and biomedical research: IEEE Xplore, ACM Digital Library, ScienceDirect, SpringerLink, Web of Science, and PubMed.

### 2.3 Search strategy

Searches conducted between February and July 2026 used Boolean expressions reflecting non-contact vital-sign and biomarker monitoring. The search returned 1,812 records; 1,251 non-journal or short papers were removed, leaving 524 records for screening. The following Boolean search expression was applied to all databases.

‘‘(non-contact vital sign monitoring) OR (contactless vital sign

monitoring) OR (non-contact physiological biomarker)’’

For IEEE Xplore and ScienceDirect, searches targeted title, abstract, and keyword fields. The ACM Digital Library search was restricted to the Digital Library collection with publication date filters applied.

### 2.4 Inclusion and exclusion criteria

Table 3 presents the inclusion and exclusion criteria. Eligible studies were English-language, peer-reviewed journal articles with full-text availability. Conference papers, reviews, editorials, non-peer-reviewed content, and studies lacking methodological detail were excluded. After title and abstract screening, 222 articles remained. Based on full-text inclusion and exclusion criteria, a further 54 articles were removed, leaving 168 studies for quality assessment. Finally, total 88 articles were selected for this systematic review. The selection process is shown in Figure 1.

**Table 3:** Criteria for inclusion and exclusion in the literature search.

Inclusion Criteria	Exclusion Criteria
Articles written in English	Non-peer-reviewed gray literature
Publications from 2019 to 2026	Repeated or duplicate studies
Research focused on non-contact vital sign monitoring	Studies lacking full-text access or limited to abstracts
Original research articles from reputed journals	Non-journal articles

**Figure 1:**
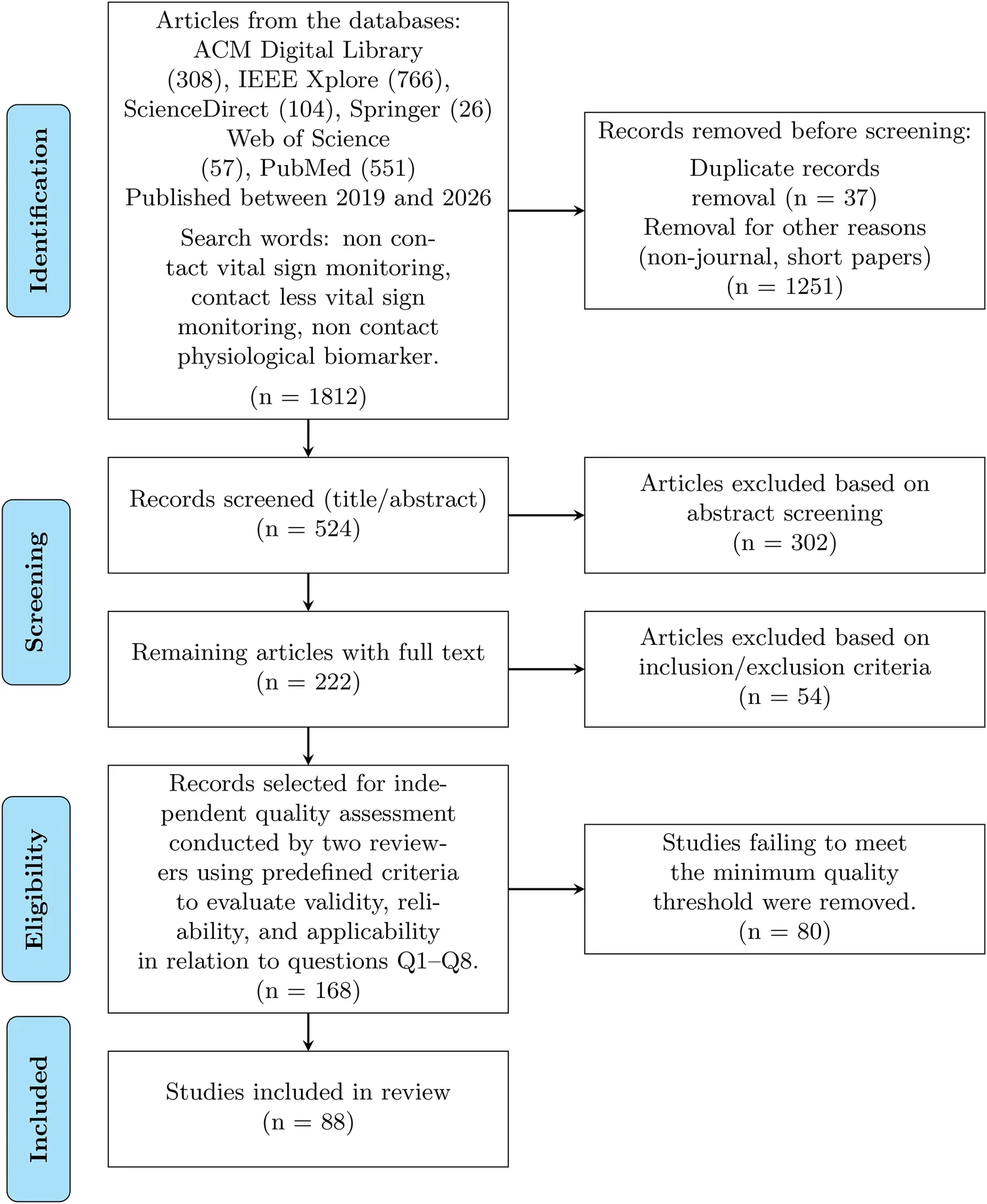
PRISMA flow chart illustrating the selection process.

### 2.5 Quality assessment

Quality appraisal followed an eight-question checklist adapted from (25–27), with each item scored as 0 (no), 1 (partially), or 2 (yes), as outlined in Table 4. Established instruments were considered but were not directly applicable: AMSTAR-2 (28) and ROBIS (29) appraise systematic reviews rather than primary studies, while QUADAS-2 (30) presumes an index test evaluated against a reference standard for a defined target condition, whereas most included studies estimate continuous physiological parameters rather than classify a diagnostic state. The checklist nevertheless aligns with recognised risk-of-bias constructs: Q2 corresponds to the QUADAS-2 *reference standard* domain, Q4 to *patient selection*, and Q3 and Q5 to *applicability*. It is therefore partitioned into two subscales such as **methodological rigour** (Q1–Q4, maximum 8) and **reporting completeness and applicability** (Q5–Q8, maximum 8). Two reviewers independently scored each study, with disagreements resolved by consensus. The retention threshold of ≥8 of a possible 16 corresponds to 50% of the maximum attainable score, a conventional midpoint cutoff in Kitchenham-derived appraisals (25). This excluded 80 of 168 appraised studies (47.6%), leaving 88 for synthesis with a mean score of 10.82/16 (range 8–14). Detailed results are provided in Table A1 in Supplementary Appendix A.

**Table 4:** Quality assessment chart for selection of studies. Items are grouped into two subscales: methodological rigour (Q1–Q4) and reporting completeness and applicability (Q5–Q8), each scored out of a maximum of 8.

Subscale	Assessment Criteria	Questions	2	0	1
**Methodological rigour** (Q1–Q4; max. 8)	Validity	Q1. Do the methods provide accurate vital sign measurements without physical contact?	Yes ◻	No ◻	Partially Yes ◻
		Q2. Are the devices validated against established clinical monitoring equipment? *(QUADAS-2: reference standard)*	Yes ◻	No ◻	Partially Yes ◻
	Reliability	Q3. Are the measurements consistent in different environmental conditions (e.g., lighting, room temperature)? *(QUADAS-2: applicability)*	Yes ◻	No ◻	Partially Yes ◻
		Q4. Is the technology reliable in diverse populations (e.g., elderly, infants, patients with movement disorders)? *(QUADAS-2: patient selection)*	Yes ◻	No ◻	Partially Yes ◻
**Reporting and applicability** (Q5–Q8; max. 8)	Applicability	Q5. Does the technology enable contact-free vital sign monitoring for patient comfort and hygiene?	Yes ◻	No ◻	Partially Yes ◻
		Q6. Does the study identify areas where it can create significant impact?	Yes ◻	No ◻	Partially Yes ◻
		Q7. Does the project/study acknowledge potential limitations or barriers?	Yes ◻	No ◻	Partially Yes ◻
		Q8. Does the study address patient privacy or data security in remote monitoring applications?	Yes ◻	No ◻	Partially Yes ◻

### 2.6 Data extraction and synthesis

Data extraction was performed independently by two reviewers using a structured extraction template. Extracted variables included sensing or system design characteristics, reported opportunities, and documented challenges. Owing to methodological heterogeneity among studies, a narrative synthesis was conducted and mapped directly to RQ1–RQ3.

## 3 Thematic analysis of the RQs

### 3.1 RQ1: which system designs and techniques are employed for non-contact vital sign monitoring?

Non-contact based vital sign monitoring is an emerging field that leverages advanced technologies to measure physiological parameters without direct skin contact. This approach is particularly beneficial for continuous health monitoring, offering comfort and convenience in various settings. The methods and system designs for non-contact monitoring are diverse, utilizing technologies such as video analysis, radar systems, sound-based techniques, Wi-Fi based techniques and so on. We aim to explore all these system designs and techniques comprehensively to address RQ1.

The signal models underlying each modality are formalised in Equations (1–20), covering radar, sound, Wi–Fi, vision, sensor, and mixed systems, respectively.

#### 3.1.1 Radar based system

In non-contact based vital sign monitoring, radar technology plays a significant role in measuring physiological parameters such as HR, RR, breathing patterns, pulse rate, and BP. Over time, radar-based monitoring systems have seen significant advancements, driven by various radar types and signal processing techniques. These developments make radar particularly advantageous in medical and privacy-sensitive environments, as they allow for monitoring without physical contact. As illustrated in Table 5, radar-based technology is commonly used for contact-free vital sign monitoring, with several radar methods emerging as the most popular choices. FMCW radar, impulse radar, MMwave radar, UWB radar, and Doppler radar are among the leading radar architectures employed for these applications. The categories (FMCW, IR-UWB, and CW-Doppler) and a workflow of radar-based technology are illustrated in Figure 2 to address RQ1.

**Table 5:** Opportunities and challenges of radar-based vital sign monitoring systems.

Ref.	Database	Vital Sign	Methods	Quantitative Performance	Opportunities	Challenges
(84)	IEEE Xplore	HR, RR	77 GHz mmWave FMCW	RR MAE: 0.39 BRPM; HR MAE: 3.64 BPM; RR R${}^{2}$ =0.98, HR R${}^{2}$ =0.72;	Multi-subject real-time monitoring; Portable hardware; Non-invasive	Distance/azimuth/posture sensitivity; Motion artifacts; 15${}^{\circ }$ angle resolution limit
(11)	IEEE Xplore	HR, RR	FMCW radar	MAE: 1.18(HR)/0.69(RR), RMSE: 1.87(HR)/1.01(RR)	Multisubject monitoring; improved localization; pandemic-ready deployment	Signal interference; I/Q imbalance; noise sensitivity
(6)	ScienceDirect	HR, RR, BP	FMCW radar	Accuracy(%): 67–100	Proactive monitoring; compact design; improved frequency use	Interference issues; high processing complexity; environmental constraints
(9)	IEEE Xplore	HR	FMCW radar	Accuracy(%): <96, RMSE: 2.85–4.35	Adaptive tracking; high accuracy; real-time capability	Noise interference; limited range; high system complexity
(35)	IEEE Xplore	HR, RR	FMCW + EEMD	RMSE: 3–6(HR)	Medical/military/rescue use; reduced bias; non-contact sensing	Weak heartbeat extraction; posture variability; reflection inconsistency
(85)	ScienceDirect	HR,RR, IBI,HRV	24 GHz radar	RMSE: 1.94(HR)/1.66(RR)	NICU suitability; reliable interval tracking; high adaptability	Cardiac/respiration separation difficulty; motion artifacts; limited validation
(45)	IEEE Xplore	RR, HRV	IR-UWB + Bi-LSTM	Accuracy(%): 82.6±6.7 in sleep stage classification	Non-contact sleep monitoring; remote sensing; improved classification	Breathing-harmonic interference; sleep-stage imbalance; signal overlap
(10)	IEEE Xplore	HR, RR	77 GHz FMCW	MAE: 2.3(HR)/0.6(RR), RMSE: 2.6(HR)/0.8(RR)	Comfortable operation; privacy preservation; lower estimation error	Clutter dominance; harmonic interference; system design complexity
(8)	IEEE Xplore	HR, RR	77 Hz FMCW	Accuracy(%): 80(HR)/94(RR)	Chronic-monitoring potential; multi-target tracking; non-contact functionality	Low SNR; untested low-error robustness; hardware non-linearities
(16)	IEEE Xplore	HR, RR	FMCW + M-Rife	–	Real-time monitoring; enhanced accuracy; non-contact measurement	Limited data volume; resolution constraints; motion interference
(86)	IEEE Xplore	RR	Doppler + monopulse	Accuracy(%): 93	Concurrent monitoring; improved accuracy; scalable architecture	Subject separation difficulty; angular resolution limits; beamwidth constraints
(50)	IEEE Xplore	HRV	24 GHz CW Doppler	RMSRE(%): 1.54, MRE(%): 1.97	Multi-target support; real-time operation; non-contact sensing	Interference sensitivity; acquisition window limits; power constraints
(87)	IEEE Xplore	RR, HR	UWB radar	–	Simultaneous HR/RR measurement; accuracy improvement;	Signal overlap; clutter noise; static reflection issues
(46)	IEEE Xplore	RR, HR	IR-UWB + CSC	Average error(%): 4.5(HR)	Improved accuracy; multi-target capability; enhanced reconstruction	Signal distortion; small datasets; multi-target interference
(51)	IEEE Xplore	RR, PR	CW Doppler	Accuracy(%): 89.4	Long-term monitoring; high-frequency resolution; versatile sensing	Noise interference; unreliable in noisy settings; challenging real-time execution
(88)	IEEE Xplore	RR, HR	77 GHz LFMCW	Error rate(%)-closer: 1.02(HR)/1.91(RR), Error rate(%)-farther: 1.31(HR)/2.12(RR)	Multi-target monitoring; improved accuracy; non-contact tracking	Random body motion; gate-crossing issues; weak signal return
(89)	ACM	HR	mmWave technology	Error(%): P-wave at 0.67, T-wave at 0.71, and QRS complex at 0.49	High-definition cardiac sensing; improved comfort; reduced clinical waste	Complex EM modeling; fidelity challenges; cross-condition variability
(90)	IEEE	BP	RSD-Net	PCC: 0.838(SBP)/0.797(DBP), ME: −0.32±6.14 mmHg(SBP)/−0.20±5.50 mmHg(DBP), MAE: 4.86 mmHg(SBP)/4.42 mmHg(DBP), RMSE: 6.14 mmHg(SBP)/5.50 mmHg(DBP)	Enhanced BP feature extraction; non-contact sensing; ML-based improvements	Signal instability; reliability concerns; limited training data
(91)	ACM	HR, RR	MSP + TM decomposition	Stationary Subjects: Error(%): 6.3(RR)/11.9(HR), PCC : 0.85(RR), Micro-RBMs: Error(%): 13.5(RR)/13.6(HR), PCC : 0.76(RR), Baseline Error: ∼20%, Baseline PCC: ∼0.6	Continuous-monitoring suitability; multi-scatter modeling; improved decomposition	Noise sensitivity; background interference; algorithmic dependence
(92)	ACM	HR, RR	Handheld mmWave + reflectors	Error(bpm): 0.11(RR) / 3.68(HR), Improvement over Baselines: Reduction(%): 38.9(RR) / 77.6(HR), Real-World Performance: RR<0.15 bpm, HR<4.16 bpm	Practical mobile sensing; improved accuracy; emergency-care potential	Motion-induced artifacts; reflector complexity; deployment constraints
(36)	ACM	RR	FMCW + FT-CNN	Laboratory Environment: Accuracy(%): 91.8, Realistic Scenarios: Accuracy: 87.3%, Experimental: Accuracy(%): 94.1, Real Vehicle: Accuracy(%): 89.3, Recognition Rate: 87.3%	Improved denoising; DL-based enhancement; better RR extraction	Environmental noise; algorithm sensitivity; real-world variability
(93)	ACM	RR	RF-GymCare + BSS	Median Similarity: 0.75, Median error(RPM):0.5(RR)	Enhanced BSS performance; better respiration separation; gym-specific sensing	Motion–respiration mixing; unpredictable movement patterns; contamination effects
(94)	IEEE Xplore	RR, HR	RF + KNN	Accuracy(%) 91	Non-invasive sensing; improved comfort; low hardware complexity	External RF noise; interference susceptibility; reduced dynamic accuracy
(34)	IEEE Xplore	HR	FMCW radar	MAE:<4 BPM, Average: 1.281 BPM, MAPE:<5%, Average: 1.735%, Accuracy(%): 98	Cost-effective HR tracking; non-contact measurement; accurate short-range sensing	Physiological interference; contamination effects; hardware constraints
(39)	IEEE Xplore	RR, HR	FMCW radar	RMSE (BPM): 4.09 (HR), Accuracy(%): 95.88	Moving-target capability; high measurement accuracy; FMCW robustness	Right-side bias; multi-target confusion; long-duration overlap issues
(95)	IEEE Xplore	RR,HR, HRV	IWR1843 mmWave	Accuracy(%): 96.5, average relative error(%): 2.7, RMSE(%):2.66, MRE(%):2.7%	High accuracy; multi-parameter sensing; clinical potential	Stationary-target interference; harmonic influence; environmental/ethical concerns
(96)	Springer	RR, HR	IR-UWB	CCC: 0.925(RR), CCC: 0.749(HR)	Clinical; multi-environment non-contact accuracy	Posture sensitivity; motion effects; limited data.
(49)	Web of Science	HR, RR	24 GHz K-band CW Doppler	RRMSE(%): 0.52–0.98 (6 subjects); Absolute error(BPM): 0.31±0.26 to 0.94±0.67; p<0.001; RRMSE<5% up to 3.2 m, <1% at 1 m; DAQ SNR: 101 dB	Beat-level resolution; virtual stethoscope; apnea detection; 3.2 m range	Bulky hardware; vibration-sensitive; range-limited accuracy; small cohort (n=6); reflective surfaces
(48)	Web of Science	RR	60 GHz FMCW radar	RR error (breaths/min), before vs after compensation – left turn: 1.801±2.777 vs 0.847±0.987; right turn: 1.822±1.849 vs 1.264±1.347; lane change: 1.336±1.647 vs 0.673±0.828; ANOVA p = 0.0003–0.0372	In-seat radar; motion index; IMU-validated	Offline only; n=3; simulated rig; RR-only; untuned threshold
(97)	Web of Science	HR, RR	Dual-antenna CW Doppler radar	–	Multi-person tracking; motion rejection; identity persistence; two-antenna setup	Moderate-motion limit; tracking-only during vigorous motion; small cohort; restricted data access
(98)	Web of Science	HR, RR	24 GHz CW Doppler radar	n=10 vs ECG/belt; quality classification 93.2–100%; RR error 0–1 bpm; HR error 0–4 bpm; hospital validation	Quality gating; ward-validated; deterioration alerting	Segment discard; small healthy cohort; 4 bpm HR error; hand-crafted features
(44)	PubMed	HR, RR	24.0–24.24 GHz CW radar	n=315 ED patients; RR bias −0.43±1.17 brpm, CCC = 0.965, RMSE 1.25; HR bias 0.50±2.09 bpm, CCC=0.986, RMSE 2.14, MAPE 1.45%	Multicentre triage; infection control; mobile point-of-care	30% data loss; low-acuity only; 150 s stillness; HR/RR only; unapproved device
(38)	PubMed	HR, RR	77 GHz FMCW	n=7; RR RMSE 0.79 brpm (96.05%), HR RMSE 2.36 bpm (90.0%); bias −0.0089/−0.0216 Hz; ADMM 0.06 s / 7.89 MB vs. 32.74 s/171.24 MB	Gridless estimation; real-time, low-memory; IoMT monitoring	SNR-limited (<−9 dB); angle-sensitive ($>30^{\circ }$); static subjects; small homogeneous cohort
(47)	PubMed	RR	7.25–10.2 GHz IR-UWB	n=3; >94.5% at 1.5 m (all angles); UR-Adjust +7.6%/+7.65%; MSE 1.23 vs. 3.7; daily-life 92.8% (30 min), 94.2% (12 h); range ≈2.77×	Free-movement RR; unconstrained daily monitoring; solitary-living safety	Single-subject; motion-discontinuity failure; range-limited (83.7% at 5 m); servo dependency; no DL baseline
(33)	PubMed	HR, RR	120 GHz narrow-beam FMCW mmWave radar	n=8; RR within ±2 breaths 90.48%; HR accuracy 90.54%	Narrow beam (high SCR); clothing penetration; portable; simplified processing	Stillness required; jitter-sensitive; distance-sensitive; small undocumented cohort; no bias/LOA
(41)	PubMed	HR, RR	60 GHz FMCW MIMO	n=5; SNR +20 dB, SINR +22 dB over CW Doppler; simulated gain 9.08 dB + 6.38 dB; with interferer RR 90.0–95.8%, HR 93.7–96.8% vs. 4.1–80.2% (Doppler)	Through-bed sensing; interferer suppression; validated signal model	Small cohort (n=5); coarse angular resolution (29${}^{\circ }$); posture-dependent; noisy heartbeat waveform; short range (≈20 cm)
(40)	PubMed	HR, RR	7 7 GHz FMCW	Range MAE −40%; ranging ≈98% (single/dual); HR accuracy 83% (vs. 46%/57%); rest RR MRE 4.47%/RMSE 1.054, HR MRE 4.60%/RMSE 3.45; field error ≈4%	Clutter-robust bin selection; tuning-free; low latency; multi-target validated	RR degradation (19.95%) under irregular breathing; empirical harmonic bounds; post-filtering needed; short range (0.2–2 m); small undocumented cohort; no public data
(37)	PubMed	HR (RR secondary)	77–81 GHz FMCW	n=10, >36,000 frames; HR MAE <4 bpm; accuracy 96.68% (A-VMD) vs. 94.66% (EWT), 89.20% (band-pass); RR error 2.11%; <5% over 0.40–1.50 m; Bland–Altman reported	Parameter-free VMD; no modal aliasing; multi-activity and multi-obstacle testing; robust post-exercise	Narrow optimum (≈0.6 m); metal degradation (MAE ≈14 bpm); WOA cost limits real-time; small narrow-age cohort; no large motion; consumer-grade references
(43)	PubMed	BP	Dual 77/79 GHz FMCW	n=5 (250 samples); ANN PWV + AUC RMSE 3.33/3.14 mmHg (SBP/DBP); polynomial 3.52/3.36; single-feature AUC → SBP 3.60, PWV → DBP 3.69	Cuff-less continuous BP; morphology-free; single-radar capable; home deployment	Dual-radar setup; per-subject calibration; tiny cohort (n=5); outlier degradation (DBP 6.24 mmHg); unresolved diastolic peak; weak wrist pulse; motion noise; no external validation
(42)	PubMed	RR	60 GHz SFCW MIMO	RR MAE 0.06 cpm (prone), 0.33 (supine), 0.80 (lateral)	3-D region-resolved imaging; privacy-preserving; blanket-tolerant; no identity leakage	Placement-dependent; misses small limb motion; high data load (≈100 GB/h); no real-time evaluation
(32)	PubMed	HR (multi-target)	77 GHz FMCW TDM-MIMO	HR MAE 0.95 bpm (single); 1.02 (separated), 1.91 (25${}^{\circ }$ apart), 2.54 (occluded), 1.88 (three subjects)	Occlusion handling; closely spaced separation; blind target counting (DBSCAN)	Seated stationary subjects; radar-facing only; steady breathing required; motion unhandled
(52)	PubMed	RR; HR (pulse)	12 GHz CW VNA-based two-antenna radar	Gain 17.8 dBi simulated / 17.06 dBi measured (<0.8 dB deviation); $S_{11}=-26$ dB at 12 GHz; bandwidth 198.5 MHz; efficiency ≈74%	Antenna-focused ablation; four topologies compared under identical conditions	Single subject; single posture; fixed 1 m range; shielded anechoic setting (100 dB absorber)

**Figure 2:**
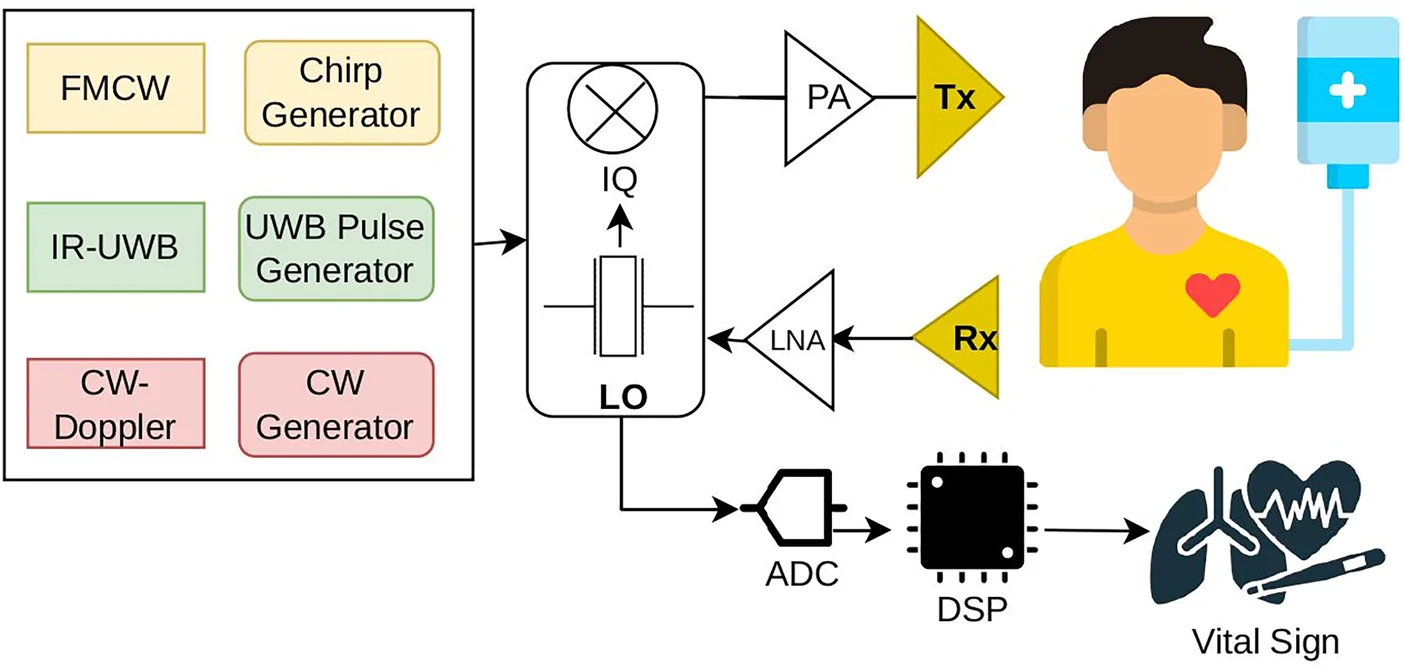
Radar based system. FMCW, IR-UWB, and CW-Doppler architectures differ only in signal generation and share a common chain: the signal is transmitted, the chest reflection received and downconverted, then digitised and processed to extract vital signs. (Tx: Transmitting Antenna, Rx: Receiving Antenna, PA: Power Amplifier, LNA: Low Noise Amplifier, IQ: In-Phase and Quadrature Mixer, LO: Local Oscillator, ADC: Analog-to-Digital Converter, DSP: Digital Signal Processor.)

Radar-based vital sign monitoring relies on detecting minute chest-wall movements caused by respiration and heartbeat. These periodic displacements modulate the phase of the received radar signal, leading to a real-valued cosine model as shown in Equation 1 (31).

$$R(t)=B\cos\left(\Omega_{c}t+\frac{4\pi }{\Lambda }\left[P\cos(\Omega_{b}t)+Q\cos(\Omega_{h}t)\right]\right)$$ (1)

Here, P and Q represent the amplitudes of breathing and heartbeat motions, while $\Omega_{b}$ and $\Omega_{h}$ denote their angular frequencies.

The operation of **FMCW** radar involves transmitting a continuous wave signal with frequency modulation over time. Vital signs are recovered from the frequency and phase difference between the transmitted and reflected waveforms, which jointly encode target range and the sub-millimetre chest displacement caused by RR and HR (8). Because range and phase are estimated together, subjects can be separated in the range domain, so more than one person can be monitored simultaneously (6, 32). Reported systems span the 24–120 GHz mmWave bands, where the shorter wavelength increases displacement sensitivity (33).

Most FMCW work concentrates on isolating the weak cardiac component from the far stronger respiratory motion and its harmonics. It uses adaptive notch filters and empirical wavelet transforms to suppress respiratory harmonics, which helps to improve the SNR (34). Related enhancement and phase-extraction schemes have further refined HR estimation accuracy (9, 10, 16, 35–37). Gridless super-resolution estimation has been proposed specifically for IoMT deployments, avoiding the quantisation error of grid-based spectral methods (38). Moreover, FMCW radar proves effective even when monitoring moving subjects. It maintains high accuracy in HR measurements, even during walking or other motion (39), and dedicated clutter-rejection schemes extend this robustness to complex multipath environments (40). Beamforming and MIMO architectures add angular selectivity on top of range resolution, enabling monitoring of a patient lying on a surgical bed (41) and improved separation of co-located subjects (42). Beyond HR and RR, mmWave FMCW radar has been used to estimate BP remotely from pulse-related waveform features (43), and remote radar acquisition has been evaluated for triage of emergency department patients (44).

Impulse radar emits short bursts (or pulses) of electromagnetic energy and measures the time it takes for the pulse to reflect back from the target. Impulse radar, specifically **IR-UWB** radar, is a promising technology for non-contact vital sign monitoring due to its high resolution and penetration capabilities. IR-UWB radar is a short-range wireless communication technology operating in the 3.1–10.6 GHz range, known for its high-speed communication and strong material penetration capabilities (45). It offers advantages like robustness to multipath propagation and high spatial resolution, making it suitable for applications such as non-contact vital sign monitoring. The study (46) involves using IR-UWB radar to detect and monitor vital signs by transmitting pulses and receiving echo signals, which are then processed to extract HR using convolutional sparse coding and noise-assisted methods. Ubiquitous respiration sensing has also been demonstrated across varying subject positions and orientations (47). Motion remains the dominant error source in practical deployments; movement-compensated processing has been used to recover RR from drivers inside a moving vehicle (48).

Unlike traditional contact-based methods, which often require electrodes or sensors attached to the body, **CW Doppler** radar provides a convenient and non-invasive alternative. These systems detect periodic movements associated with cardio-pulmonary activities, making them suitable for applications in both clinical and home settings (49).

The study by Petrović et al. (50) employs a 24 GHz CW Doppler radar with quadrature architecture for non-contact HR measurement. This system uses a single-frequency wave modulated by the target’s movement, enabling the detection of relative displacement. However, any movement within the radar’s field of view affects the measurement, posing challenges in monitoring multiple targets simultaneously, since the transmitted single-tone signal carries no range information with which to separate targets. Additionally, Goto et al. (51) and Petrović et al. (50) highlight the effectiveness of CW Doppler radar technology in vital sign monitoring, reinforcing its potential as a versatile tool for non-contact physiological assessments. Phase-based variants extend this principle to transmission measurements, with a 12 GHz high-gain patch array recovering vital signs from the phase of the $S_{21}$ coefficient (52).

#### 3.1.2 Sound based system

Sound-based system represents a promising approach in non-contact vital sign monitoring, leveraging sound waves for precise physiological measurements. Figure 3 illustrates two types of sound-based system design (ultrasound and acoustic) along with workflow utilized by researchers over the past five years. Unlike microwave radar systems that use electromagnetic waves, sound-based systems benefit from lower-cost hardware and reduced susceptibility to electromagnetic interference.

**Figure 3:**
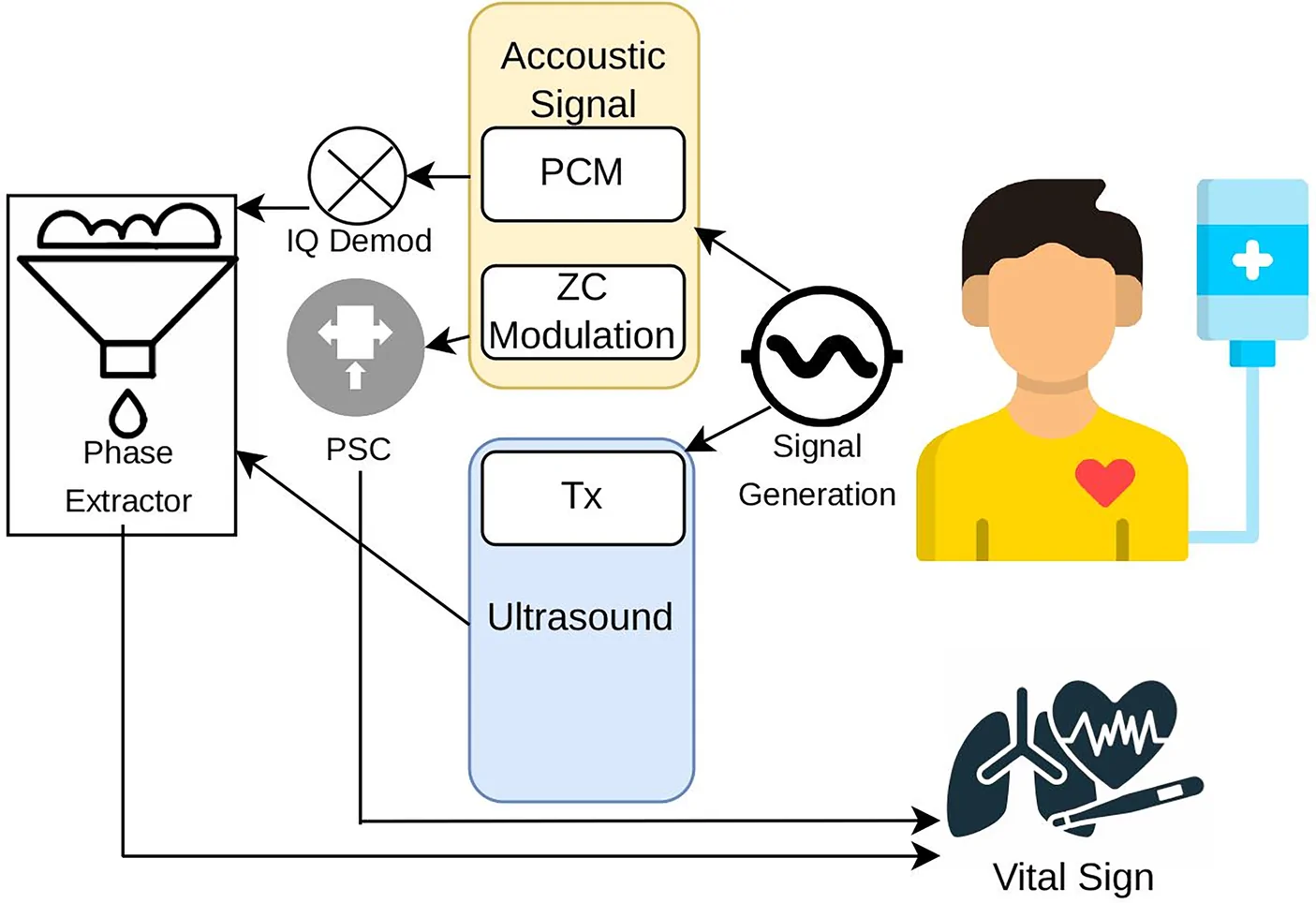
Sound based system. Two types of signals (ultrasound and audible-band acoustic) are used, each generated and modulated before transmission. The echo returning from the chest is demodulated and its phase tracked, and the resulting displacement waveform yields the vital signs. (IQ Demod: In-Phase and Quadrature Demodulation, PCM: Pulse-code Modulation, PSC: Path Selection and Combination.)

In sound-based vital sign monitoring systems, including ultrasound (53, 54) and active acoustic sensing with commodity speakers and microphones (55, 56), a probing signal $s_{\text{tx}}(t)$ is transmitted and the received signal can be modeled as

$$x(t)=\sum_{k=1}^{K}\alpha_{k}s_{\text{tx}}\left(t-\tau_{k}(t)\right)+n(t),$$ (2)

where $\alpha_{k}$ is the complex attenuation associated with user k, and

$$\tau_{k}(t)=\tau_{0,k}+\frac{2Y_{k}(t)}{c_{s}}$$ (3)

is the time-varying delay, with $\tau_{0,k}$ the static propagation delay determined by the nominal distance to user k, $c_{s}$ the speed of sound, and $Y_{k}(t)$ the chest displacement due to vital signs. For RR and HR, the displacement of user k can be expressed as

$$Y_{k}(t)=P_{k}\cos(\Omega_{b,k}t)+Q_{k}\cos(\Omega_{h,k}t),$$ (4)

where $P_{k}$ and $Q_{k}$ denote the amplitudes of the respiratory and cardiac motions, and $\Omega_{b,k}$ and $\Omega_{h,k}$ are their corresponding angular frequencies. The term n(t) represents additive noise and unmodeled reflections.

Ultrasound-based vital sign monitoring uses high-frequency sound waves to measure physiological parameters such as HR, RR, and BP. For example, the system in Ambrosanio et al. (53) detects HR using ultrasound. The continuous-monitoring setup employs a 40 kHz cosinusoidal signal, suitable for tracking skin displacements up to 4 mm. To reduce phase-wrap artifacts from larger movements, four receiving transducers are used to capture diverse signal paths. A voltage-controlled oscillator generates the 40 kHz signal, which is shaped and processed using an XOR-based phase detector to extract phase shifts between transmitted and received signals. The multi-channel design prevents null points instances where, overlapping signals hinder detection by selecting the most reliable channel at any given time. Because simultaneous nulls across all channels are unlikely, the system ensures robust and continuous vital-sign monitoring. Beyond averaged HR, air-coupled ultrasound has been shown to resolve individual beat intervals with sufficient temporal precision to derive HRV, and its accuracy has been characterised against reference measurements under varying subject posture and sensor placement (54). This extension is relevant to IoMT applications, since HRV rather than mean HR is the parameter of interest in autonomic and stress-related assessment.

SonarBeat (55) is a sound-based system that uses phase-based active sonar to monitor respiration rates with a standard smartphone. It operates in the inaudible 18–22 kHz range, transmitting acoustic signals that reflect off the chest and are received by the phone’s microphone. These reflections are processed using STFT and a multi-stage pipeline including signal generation, data extraction, and respiration estimation, to enable accurate, real-time monitoring for a single user.

RespTracker (56) extends sound-based monitoring to multi-user environments. It employs a signal model with a two-stage path selection and user separation process, identifying candidate paths through respiration SNR analysis and distinguishing users via distance (CIR) and angle (beamforming) features. A two-round path combination mechanism reconstructs high-quality respiration signals by clustering relevant paths. The system is evaluated under noise conditions such as music, speech, and white noise, demonstrating robust performance using metrics like BrPM and breath interval time. While SonarBeat (55) is effective for single-person monitoring, RespTracker (56) provides a scalable solution for continuous, room-scale respiration tracking in complex domestic settings despite challenges such as sound attenuation.

Acoustic sensing has also been applied to screening rather than continuous monitoring alone. A pneumoconiosis monitoring and warning system uses acoustic signals to characterise abnormal respiratory patterns and flag at-risk individuals in occupational settings (57), illustrating that the respiratory waveform recovered by sound-based sensing carries diagnostic information beyond the RR value itself.

Table 6 summarizes existing research on sound-based technologies for contactless vital sign monitoring, highlighting the opportunities and challenges in this field.

**Table 6:** Opportunities and challenges of sound-based vital sign monitoring systems.

Ref.	Database	Vital Sign	Methods	Quantitative Performance	Opportunities	Challenges
(53)	IEEE Xplore	HR, RR, HRV	CW architecture(40 kHz)	MAE(BPM): 0.02	Low-cost; safe acoustic sensing	Null-point issues ; breathing–pressure–motion interference.
(56)	ACM	RR	RespTracker system(48 kHz with 32-bit float precision)	Error(BPM): 0.6 (single user), 0.8 (multiple user)	Range limitation from air attenuation	Domestic sensing potential with improved algorithm efficiency.
(55)	ACM	RR	SonarBeat System	Median error(BPM): 0.2	Short-range; single-person effectiveness	Static-vector issues; motion/environment sensitivity; limited performance under blanket
(57)	PubMed	RR	$P^{3}$Warning: inaudible chirp (18–22 kHz), COTS speaker/mic	Median RR error 0.39 bpm	Low-cost COTS hardware; noise-immune (<14 kHz); mine-tunnel validated; occupational screening	Single-target; static subject required; angle-limited (>60${}^{\circ }$); body-size sensitive; screening only
(54)	PubMed	HR, HRV, RR	Air-ultrasound ToF, 100 kHz (piezo pair, 0.5 m, PRF 250 Hz)	IBI MAE 1.5–11 ms (best 1.34 ms); correction −30% MAE, −24% SD; SDNN/RMSSD near ECG	Micrometre sensing; BCG/SCG-comparable; infection control	Calibration-dependent; ECG-dependent; empirical targeting; tiny cohort (n=4); error-sensitive HRV

#### 3.1.3 Wi-Fi based system

Wi-Fi based non-contact vital sign monitoring leverages existing wireless communication technologies to measure physiological parameters such as HR, RR, and even body movement without the need for physical contact. This approach uses the reflection and distortion of Wi-Fi signals caused by a subject’s physiological activities to extract vital sign information. A practical advantage over dedicated radar or ultrasound hardware is that the sensing infrastructure is already deployed in most indoor environments, so monitoring can in principle be added without installing new devices (58).

WiFi-based vital sign monitoring models chest-induced motion as small, time-varying perturbations in the wireless channel. The CSI/CFR for subcarrier $f_{n}$ can be expressed as

$$H(f_{n},t)=\sum_{k=1}^{K}\alpha_{k}(f_{n})e^{-j2\pi f_{n}\tau_{k}(t)}+W(f_{n},t),$$ (5)

where $\alpha_{k}(f_{n})$ is the complex gain of path k and $W(f_{n},t)$ denotes noise. The delay variation caused by respiration and heartbeat is

$$\tau_{k}(t)=\tau_{0,k}+\frac{2Y_{k}(t)}{c},$$ (6)

with chest displacement modeled as

$$Y_{k}(t)=A_{b,k}\cos(\Omega_{b,k}t)+A_{h,k}\cos(\Omega_{h,k}t).$$ (7)

In CIR-based systems such as Guan et al. (59), the time-domain channel is written as

$$h(\tau ,t)=\sum_{k=1}^{K}\alpha_{k}\delta \left(\tau -\tau_{k}(t)\right),$$ (8)

and relates to the CFR through

$$H(f,t)=\int h(\tau ,t)e^{-j2\pi f\tau }\;d\tau .$$ (9)

For mmWave systems such as ViMo (7), a simplified phase model is used:

$$H(f_{n},t)=H_{0}(f_{n})e^{\;j\frac{4\pi }{\lambda }Y(t)}+W(f_{n},t),$$ (10)

where the phase term captures fine chest-wall movements.

In the SpaceBeat system (60), **CSI** is used to monitor vital signs by capturing Wi-Fi signal variations caused by respiration and heartbeat. The system collects CSI from 30 OFDM subcarriers and analyzes phase variations to clean the raw measurements, removing irrelevant distortions. It then performs 2D AoA analysis with multiple antennas to obtain spatial information for locating individuals and separating their signals. To suppress noise, a harmonic canceller removes breathing-related interference and enhances the SNR of heartbeat components. A cPCA framework is applied to separate the target user’s vital signs from others, enabling effective noise reduction. For feature extraction, the cPCA-CL method decouples individual signals, allowing accurate estimation of breathing and heart rates. An iterative refinement strategy further improves localization and signal separation by repeatedly reapplying cPCA-CL using updated location-based datasets.

Whereas SpaceBeat targets multi-person separation, VitalCSI (58) addresses the complementary question of whether reliable RR estimation is achievable on consumer-grade Wi-Fi hardware rather than specialised or research-oriented CSI platforms. Restricting the target to respiration alone, rather than the weaker cardiac component, keeps the required SNR within what commodity chipsets can deliver. This trade-off between the range of parameters recovered and the accessibility of the hardware is a recurring design choice across Wi-Fi based systems, and is directly relevant to IoMT deployments where cost and installation burden constrain what can realistically be placed in a home.

ViMo (7) is a multiuser vital sign monitoring system that uses commercial 60 GHz Wi-Fi to estimate RRs and HRs without calibration. It captures vital signs through **CIR** measurements and employs an adaptive object detector to identify both static and moving subjects. To improve heartbeat SINR, ViMo removes breathing interference using a smoothing spline that estimates and subtracts respiration from the CIR, followed by a DP algorithm applied to the spectrogram to suppress random noise. For feature extraction, the system estimates the breathing waveform and eliminates it in the time domain, leveraging the stationarity of heart rates for accurate HR detection. This combination of adaptive detection, noise reduction, and signal processing enables ViMo to achieve high accuracy, with median errors of 0.19 BrPM for RR and 0.92 BrPM for HR. Its design supports robust monitoring across varying distances, orientations, and multiuser scenarios.

In the multi-person breathing detection system (59), **CFR** is used to remove phase offsets caused by asynchronous transceiver devices, which helps isolate breathing signals from mixed antenna inputs. The system applies beamforming to scan the environment in two dimensions and focus on specific areas, improving the detection of each person’s breathing. Static clutter is removed to clarify the signals, although the paper does not detail the exact algorithm. For feature extraction, the system combines AOA and frequency-domain analysis to form an AOA–FREQ spectrogram that shows each target’s breathing frequency and position. Clustering is then used to group features and separate individual breathing signals. This approach effectively distinguishes respiration patterns from multiple subjects and supports real-world applications such as home health monitoring.

Based on the Table 7 and the discussion above, Figure 4 illustrates an overall representation of the types of Wi-Fi-based technology along with the flow of contact-less vital sign monitoring.

**Table 7:** Opportunities and challenges of Wi-Fi-based vital sign monitoring systems.

Ref.	Database	Vital Sign	Methods	Quantitative Performance	Opportunities	Challenges
(7)	IEEE Xplore	HR, RR	ViMo (Wi-Fi CIR)	Median error(BPM): 0.19(RR), 0.92(HR)	Multiperson monitoring; high resolution and accuracy; robust signal processing	Signal attenuation with distance; interference from respiration and noise; practical deployment considerations; hardware limitations
(99)	ScienceDirect	RR, HR	Survey on Wi-Fi-CSI	Accuracy(%): one study 99, another over 95(one person),over 88 (two persons), 100(LOS). Maximum errors of less than 0.7 bpm and average errors around 0.15 bpm	Non-invasive real-time multi-person monitoring	Movement-induced interference; reliability and portability limits; standardization and scalability challenges.
(60)	ACM	RR, HR	Commodity Wi-Fi devices and CSI	Accuracy(%): 99.1(RR),97.9(HR)	Enhanced multi-person resolution in crowded environments	Vital-sign extraction complexity; localization sensitivity
(59)	IEEE Xplore	RR	Wi-Fi CFR	Accuracy(%): 97.5(single person), 97.04(multiple person)	Low-cost; rapid multi-person non-contact respiratory sensing	Signal–target mapping difficulty; environmental reliability challenges
(58)	PubMed	RR	VitalCSI (Wi-Fi CSI)	RR MAE 1.20 brpm; RMSE 2.01; $r^{2}=0.93$; bias −0.18 brpm; range 6–33 brpm	Contactless continuous sensing; low-cost COTS Wi-Fi; privacy-preserving; wide RR range; home/athletic use	Motion artifacts; CSI noise; seated single-subject protocol; multi-subject, range, through-wall unvalidated

**Figure 4:**
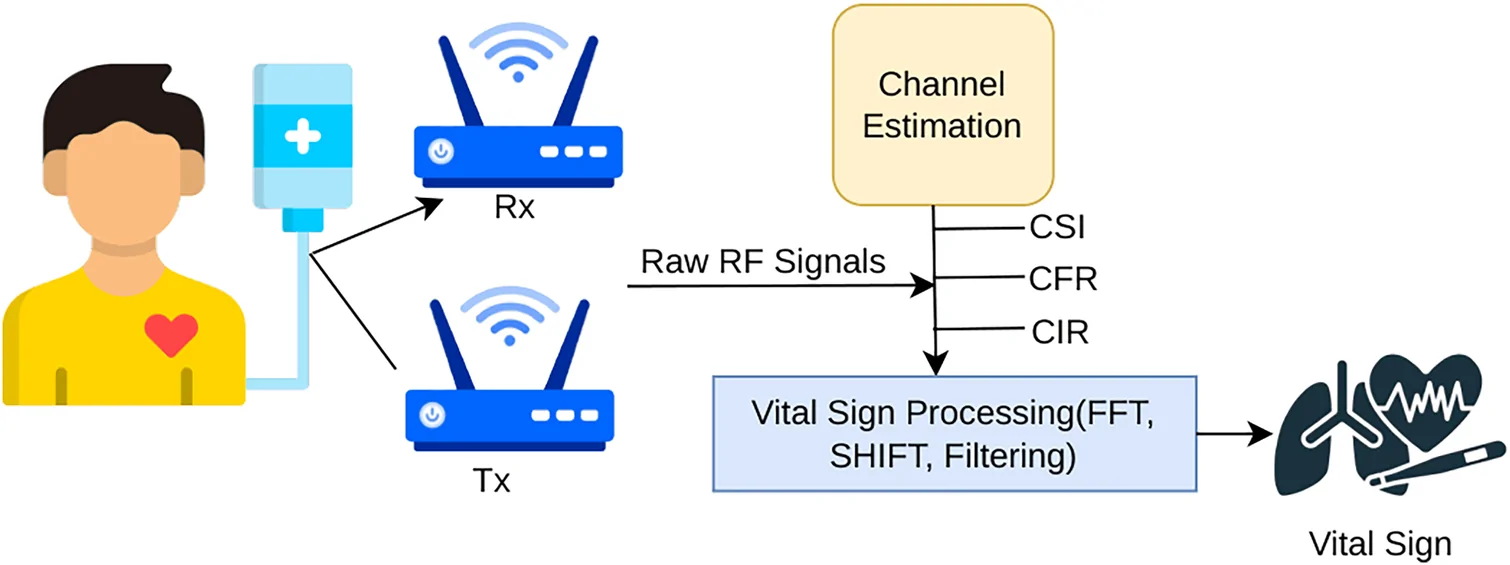
Wi-Fi based system. Respiration and heartbeat perturb the wireless channel between transmitter and receiver; the raw RF signal is passed to channel estimation, yielding a CSI, CFR, or CIR representation, from which the vital signs are recovered by frequency-domain processing.

#### 3.1.4 Vision based system

Vision-based vital sign monitoring uses imaging technologies, such as RGB cameras, to assess physiological parameters like RR, HR, and SpO${}_{2}$ without direct contact with the body. This approach leverages advances in computer vision and signal processing algorithms to extract vital signs from image or video data. It typically focuses on subtle visual cues, such as skin color variations or body movements, that reflect underlying physiological changes. The underlying sensor need not be a conventional RGB camera: thermal (61, 62) and depth (63, 64) imaging recover the same parameters from emitted infrared radiation and geometric chest-wall displacement respectively, broadening the conditions under which vision-based monitoring remains viable.

In vision-based vital sign monitoring, the observed signal can be modeled as

$$S_{m}(t)=I_{0,m}+\sum_{\ell =1}^{L}\gamma_{m,\ell }\;V_{\ell }(t)+\eta_{m}(t),$$ (11)

where $S_{m}(t)$ denotes the time-varying signal from channel m (e.g., an RGB component, a thermal channel, or a motion/displacement signal), $I_{0,m}$ is the static baseline term, and $V_{\ell }(t)$ represents latent physiological components such as respiration and heartbeat. The coefficients $\gamma_{m,\ell }$ weight the contribution of each physiological component to channel m, and $\eta_{m}(t)$ models noise due to illumination changes, motion artifacts, sensor noise, and other disturbances. For respiration and heartbeat, a common parametric form is

$$V_{\ell }(t)=A_{\ell }\cos(\Omega_{\ell }t+\phi_{\ell }),$$ (12)

where $A_{\ell }$, $\Omega_{\ell }$, and $\phi_{\ell }$ denote the amplitude, angular frequency, and phase of the ℓ-th physiological component (e.g., breathing or cardiac activity). This model generalizes the signal formulations used in prior vision-based vital sign monitoring systems (65–69), and applies equally to thermal and depth channels, where $S_{m}(t)$ corresponds to radiometric temperature or measured distance rather than pixel intensity (61, 63).

Based on the analysis of the studies in our research, there are three types of designs aligned with vision based technique. Figure 5 depicts various types of vision based techniques with the workflow.

**Figure 5:**
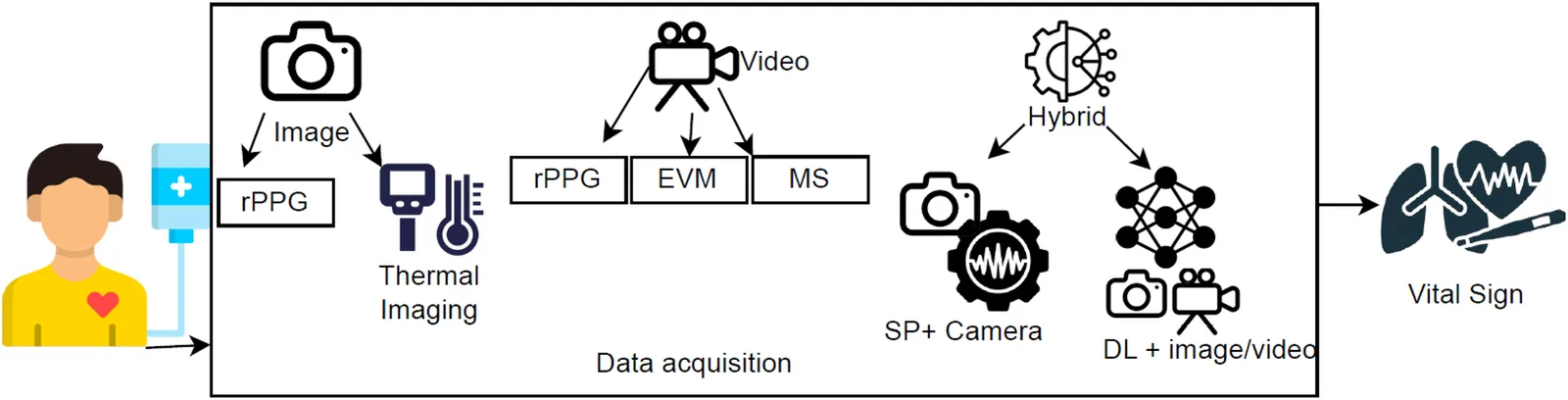
Vision based system. Three design categories are used: image-based acquisition processed by rPPG or thermal imaging, video-based acquisition processed by rPPG, EVM, or multispectral analysis, and hybrid designs pairing a camera with signal processing or deep learning. (MS: Multispectral, SP: Signal Processing, DL: Deep Learning.)

Image-based monitoring systems use still images captured from RGB, thermal, or multispectral cameras to extract vital signs by analyzing individual frames with image processing or deep learning methods. RGB-based approaches such as Mehta and Sharma (70) demonstrate a non-contact technique for estimating respiratory rate, offering a comfortable and cost-effective alternative to wearable sensors. Similarly, Maurya et al. (65) integrates RGB and thermal imaging through image registration to automatically select and track the ROI for breathing rate monitoring.

The ReViSe framework (66) extends image-based monitoring to smartphone cameras by capturing face images and extracting vital signs using rPPG. It identifies optimal ROIs for BVP signals, applies noise reduction techniques, and combines intensity-based and motion-based analysis to measure HR, SpO${}_{2}$, and BP accurately.

Within the image-based category, thermal imaging forms a distinct branch that does not depend on ambient illumination, since respiration is detected from the periodic temperature change around the nostrils and mouth caused by inhaled and exhaled air rather than from reflected light. Because this thermal contrast varies with subject, distance, and ambient temperature, adaptive signal analysis has been applied to sustain reliable RR estimation in real time (61). The illumination independence of this modality is most valuable in settings where RGB acquisition is impractical, and thermal cameras have accordingly been evaluated for overnight respiratory measurement in a clinical sleep environment (62).

Video-based monitoring systems use continuous RGB, thermal, multispectral, or hybrid video streams to track vital signs. For example, Ahani et al. (67) employs RGB video to estimate RR in infants, using EVM and an adaptive peak prominence threshold to handle low-light conditions and obstructions such as blankets. Likewise, Scebba et al. (68) introduces a multispectral fusion approach using RGB, NIR, and FIR cameras to estimate RR and detect apnea, with the S2 Fusion algorithm combining respiratory-induced motion and plethysmographic features to enhance accuracy.

Another video-based system, presented in Alnaggar et al. (69), extracts rPPG waveforms from facial video to measure vital signs. This method offers a non-contact, convenient, and accessible alternative to traditional sensor-based approaches, providing a safer and more comfortable experience for users.

Recent facial rPPG work has moved from demonstrating feasibility toward characterising measurement quality. A pilot validation study reports agreement against a reference for both averaged and quasi-instantaneous HR (71), the latter being the harder case because it requires reliable beat-to-beat peak localisation rather than a spectral average over a long window. Reducing the observation window is itself an active concern, as short acquisition times determine whether the technique is usable in triage or walk-up screening rather than only in prolonged monitoring (72). Extraction of BP from video has also been investigated (73), although this remains substantially more difficult than HR or RR, since BP is inferred indirectly from pulse waveform morphology rather than measured from a periodic component. Robustness outside controlled laboratory conditions remains the principal barrier: a systematic evaluation across challenging environments quantifies how illumination variation, subject motion, and compression artifacts degrade rPPG accuracy, and compares artifact mitigation strategies and network architectures under these conditions (74).

The hybrid approach to vision-based vital sign monitoring integrates video magnification and DL techniques to estimate vital signs such as HR and BP from facial videos. This method extracts dual-path time-series signals from a single facial video, which collaborate to improve accuracy (12). By combining image and signal processing technologies, the approach mitigates interference from factors such as skin characteristics and environmental conditions. Furthermore, a compact two-stage CNN leverages features from stable phases, ensuring precise estimations even with limited sample data.

Hybrid designs also arise when a depth camera is paired with a learned detection stage. Depth sensing recovers respiration from the geometric displacement of the chest and abdomen rather than from colour variation, which makes it insensitive to both skin tone and lighting, but it first requires the thoracic region to be located reliably. Combining AI-based body detection with depth measurement addresses this, and allows a continuous respiratory waveform to be reconstructed rather than a rate alone (63). Where the detection stage is instead handled by conventional signal processing, the same sensing principle has been assessed for clinical feasibility in an anesthesia setting, a demanding case in which drapes, equipment, and clinician movement obstruct the field of view (64). Table 8 highlights the opportunities, challenges, and methodologies employed in non-contact, vision-based vital sign monitoring.

**Table 8:** Opportunities and challenges of vision-based vital sign monitoring systems.

Ref.	Database	Vital Sign	Methods	Quantitative Performance	Opportunities	Challenges
(100)	Springer	HR, RR, SpO${}_{2}$	Azure Kinect RGB-D	HR MAE: 7.69 bpm; RR MAE: 4.83 bpm (ventilator); SpO${}_{2}$ MAE: 3.37%; TV MAE: 0.61 mL	Non-contact; no skin damage; flow-volume loops; low-cost	RGB fails in phototherapy/low light; n=3 ventilated; retrospective; visible skin required; IR interference.
(69)	ScienceDirect	HR, RR	EVM with rPPG	HR (RGB): MAE = 2.05 BPM, PCC = 0.91, HR (HSV): MAE = 2.03 BPM, PCC = 0.86, RR: MAE = 1.62 BrPM, PCC = 0.45	Safer, low-infection, telehealth-friendly	Lighting; motion; video-quality sensitive; affected by accessories.
(70)	ScienceDirect	RR	OPOIRES using RGB cameras	Cohface GL: MAE = 0.48 BPM, RMSE = 1.13 BPM, Cohface BL: MAE = 0.60 BPM, RMSE = 1.40 BPM, DEAP: MAE = 1.63 BPM, RMSE = 2.12 BPM	Comfortable; low-cost consumer-camera use	Visibility; interference; and motion limitations
(67)	IEEE Explore	RR	EVM and an adaptive peak prominence threshold with RGB video	Manual ROI: ICC = 0.91 (95% CI: 0.89–0.93), Automatic ROI: ICC = 0.88 (95% CI: 0.85–0.90)	NICU-ready; lighting-robust performance	Illumination sensitivity and RR inconsistency
(13)	IEEE Explore	HR, HRV, and RR	EVM with video	MAE: 0.50 (LED only), 3.21 (LED ceiling), 3.60 (Fluorescent Ceiling)	Lighting- and skin-robust monitoring	Non-facial sensing difficulty; frequency/thermal filter limits
(101)	ScienceDirect	RR	Intel D415 RealSense camera	RMSD accuracy of ≤1.0 breaths/min for set rates and ≤1.3 breaths/min for spontaneous breathing	Continuous; infection-safe reliability	Difficult breath detection; stable-camera dependence.
(102)	ScienceDirect	HR, RR, BP, and SpO${}_{2}$	Digital camera with rPPG	SBP (Sitting): Mean Diff = 0.78 mmHg, SE = 1.23 mmHg, SBP (Lying): Mean Diff = 5.19 mmHg, SE = 1.17 mmHg, DBP (Sitting): Mean Diff = −2.68 mmHg, SE = 1.32 mmHg, DBP (Lying): Mean Diff = 1.47 mmHg, SE = 1.27 mmHg	Contactless monitoring; integration with robotics; cross-demographic testing	Motion artifacts; ambient light variability; skin tone variation; signal processing complexity
(12)	ScienceDirect	HR, BP	Video and image processing with PCA, deep learning methods	HR: RMSE = 1.056, MAPE = 1.576%, SBP: RMSE = 4.11, MAPE = 3.73%, Test = 4.839, DBP: RMSE = 3.75, MAPE = 4.95%, Test = 4.106	Non-contact; accessible; enabled by DL.	Interference from skin; breathing; motion; lighting.
(65)	ScienceDirect	RR	RGB and thermal imaging systems	Normal Breathing: MAE = 0.11 BrPM, Phase 1: MAE = 1.46 BrPM, Phase 2: MAE = 2.08 BrPM, Phase 3: MAE = 1.69 BrPM	Sports and rehabilitation robustness	Dynamic conditions; camera setup; facial-motion challenges
(68)	IEEE Xplore	RR	Multispectral videos using RGB, NIR, and FIR cameras	RR (Monospectral): RMSE = 4.64 BrPM, RR (Multispectral): RMSE = 1.60 BrPM, Obstructive Apnea: F1 = 0.75–0.86, Central Apnea: F1 = 0.75–0.93, Independent Apnea Detection: RMSE = 4.44 BrPM (vs. 7.96)	Safe for skin, widely usable	Camera, environment, algorithm.
(103)	ScienceDirect	RR, HR, Temperature	vPPG	HR: Mean Diff = 5.9 ± 12.8 BPM, Filtered = 2.9 ± 8.1 BPM, R = 0.6833, RR: Mean Diff = −0.4 ± 2.6 BrPM, Filtered = −0.2 ± 1.2 BrPM, R = 0.8128, Temp: Mean Diff = 0.83 ± 0.67${}^{\circ }$C, R = 0.5176	Efficient triage; safer; improved workflow	Algorithm; pediatrics; temp bias.
(104)	IEEE Xplore	Temperature	Video using RGB camera	NEST-Lab: F1 = 0.81, NEST-Wild: F1 = 0.76, Small Head Deviation (<5${}^{\circ }$): F1 = 0.65, Large Head Deviation (>25${}^{\circ }$): F1 = 0.85, Wild Large Deviation: F1 = 0.78, Wild Small Deviation: F1 = 0.67	Widespread devices; real-time use	Accuracy; reliability; head motion.
(66)	IEEE Xplore	HR, HRV, SpO${}_{2}$, BP	rPPG using smartphone camera	HR (TokyoTech/PURE): MAE = 1.73 / 3.95 BPM, HRV (TokyoTech/PURE): MAE = 18.55 / 25.03 ms, SpO${}_{2}$ (PURE): MAE = 1.64%, HR (Video-HR): MAE = 2.49 BPM, SBP (Video-BP): MAE = 6.7 mmHg, DBP (Video-BP): MAE = 9.6 mmHg	Remote monitoring; daily use; commercialization	Noise; processing; datasets.
(105)	ACM	HR, RR, BP	RGB Camera	SBP: MAE = 3.59 mmHg, R = 0.61, DBP: MAE = 2.63 mmHg, R = 0.54	Telemedicine-ready monitoring	Personalized BP calibration required
(106)	Springer	HR, RR, SpO${}_{2}$, BP, Core Temperature	Video camera	HR: MAE = 2.5 BPM, Coverage = 53.2% (103 h), RR: MAE = 2.4 BrPM, Coverage = 63.1% (119.8 h)	Non-contact early post-op monitoring	Limited valid data
(107)	Web of Science	HR, RR	850 nm indirect ToF camera + depth-based motion compensation	n=29(286 measurements); HR success 75% (compensated) vs. 35%; mean HR deviation −6.25 vs. 21 bpm; RR deviation 1.7 bpm	Skin-tone independent (74% both groups); lighting-agnostic; direct chest-wall RR; low-cost consumer ToF	Depth-invisible facial motion; blink-induced ROI jitter; clothing occlusion; wide LOA (±35 bpm)
(108)	Web of Science	HR, RR	850 nm indirect ToF camera + STFT quality weighting	HR success (compensated/uncompensated): parked 86/36.5, highway passenger 82/34, highway driver 71.9/13.6, local passenger 66/22, local driver 56/12; RR deviation −1.4 bpm (71% within ±3 bpm); >125 measurements per scenario	On-road validation; single-sensor HR and RR; lighting-robust (5–1800 lux); ADAS/DMS integration	Local-road degradation; head rotation uncompensated; clothing-induced RR error (3.57 bpm); small in-house cohort
(109)	Web of Science	SpO_2_, PR, RR	Monochrome camera + time-division multiplex NIR illumination (660/810/940 nm)	n=9 piglets; SpO_2_ mean difference 0.7%, RMSE 3.8%, MAE 2.93%, LoA −6.64 to +8.02%; HR mean difference −9.193 bpm	Contactless SpO_2_; incubator-integrated; ambient-light independent (active NIR); NICU pathway	Sedated animal model; respiratory harmonic interference; motion artifacts; unfiltered non-physiological values; no clinical calibration
(63)	PubMed	RR (waveform)	Azure Kinect depth camera	Chest ROI: lowest median RMSE/MAE, highest reference correlation; small Bland–Altman bias for chest and abdominal ROIs vs. whole torso	Contactless CT-suite monitoring; early tachypnea/hyperventilation detection; waveform basis for volume surrogates	ROI-critical (not visually judgeable); clothing attenuation; noisy whole-torso signal; small seated-only cohort; supine unvalidated
(72)	PubMed	HR	Facial-video rPPG	MAE 1.50 bpm; RMSE 3.07 bpm; PCC 0.97 (COHFACE); MAE 1.97 (studio) vs. 2.21 bpm (natural lighting), not significant	Contact-free (no skin irritation); ICU-to-home deployable; stage-wise interpretability	Motion-induced artefacts; illumination-dependent ROI filtering; weak rPPG SNR under compression; harmonic rate errors
(61)	PubMed	RR	Thermal imaging	MAE 0.57 ± 0.36 BPM; RMSE 0.64 ± 0.42 BPM; stable under motion, speech, yaw, supine, to 2.0 m; 65.2 ms/frame, 22.5 FPS (Jetson Orin Nano)	Privacy-preserving; fully edge-resident (no cloud); recalibration-free adaptive processing	Nostril visibility and thermal contrast required; range-limited (>1.5–2.0 m); nasal-breathing dependent; weakened by diaphragmatic breathing or high ambient temperature
(64)	PubMed	RR	Intel D415 RealSense depth camera	RMSD 1.92 brpm, bias 0.30; by stage 1.71 (pre-), 1.95 (intra-), 2.13 (post-anaesthesia); meets 3 brpm target over 4–40 brpm	Wire-free (comfort, infection control); instant acquisition; PACU and remote multi-patient monitoring	Motion artefacts (stable periods only); warming-blanket confounding; healthy volunteers under protocol; trolley-based prototype
(62)	PubMed	RR	Thermal cameras	MAE 0.64–0.91 BPM (best time-domain); breath detection sensitivity 96.3%, precision 94.1%; IBI MAE 0.48 s	Unobtrusive (sleep-preserving); strong PSG agreement; single-camera sufficiency	Thermal noise; movement-induced camera displacement
(73)	PubMed	BP	V-BPE: pulse transit time from CHROM rPPG signals at forehead and palm in one frame	MAE 17.1 mmHg (SBP), 13.2 mmHg (DBP) vs. 36.2/29.1 (fixed-parameter baseline)	Commodity RGB cameras (phone-scalable); contact-free (infection control); demographic conditioning aids generalisation	Vessel parameters unmeasurable from video; empirical PWV assumptions; exceeds ISO accuracy limits; requires simultaneous face and hand visibility
(71)	PubMed	HR	Facial-video rPPG	MAE 0.1 bpm (60 s), 0.2 (10 s), 0.4 (4 s); errors ≤1 bpm in 100%/99.8%/94.5%; r=0.992/0.999/0.997 (p<0.001)	Telemedicine, triage, driver monitoring, biofeedback; contact-free during epidemics	Controlled lighting and fixed camera; motion and expressions untested; hardware-dependent accuracy; fixed 0.3 s alignment bias
(74)	PubMed	HR	Facial vPPG	MAE 0.1/0.2/0.4 bpm (60 s/10 s/4 s); r=0.992/0.999/0.997 (p<0.001)	Smartphone-only HR (no added hardware); telemedicine, triage, driver monitoring, biofeedback	Small homogeneous cohort; skin-tone bias; controlled lighting; motion and expressions untested; hardware-dependent results

#### 3.1.5 Sensor based system

Sensor-based non-contact vital sign monitoring systems employ various sensor technologies such as VLS, EPS, ultrasonic sensors, and capacitive sensors to gather data on vital signs like HR, RR, SpO${}_{2}$, body temperature, and BP. In non-contact sensor-based vital sign monitoring, ultrasonic, capacitive, and VLS encode physiological activity through different physical mechanisms. In ultrasonic sensing, chest-wall motion modulates the phase of a received acoustic signal, which can be modeled as

$$Rx(t)=\hat{A}\sin\left(2\pi ft+\varphi (t)\right),$$ (13)

where φ(t) reflects RR- and HR-induced displacement (75). In capacitive sensing, chest motion alters the effective sensor capacitance, expressed as

$$C(t)=C_{0}+\Delta C(t),$$ (14)

with ΔC(t) carrying the vital sign information (75, 76). For VLS, periodic chest motion modulates the received optical power according to a Lambertian channel model,

$$P_{r}(t)=K[d(t){]}^{-\gamma },$$ (15)

where d(t) is the time-varying distance between the subject and the receiver (3). Although these modalities rely on different physical principles, they all measure periodic chest displacement caused by physiological biomarkers, enabling unified vital sign estimation. This holds for optical variants more generally whether the detected quantity is received power, a coherent speckle-pattern shift, or light modulated within a fiber, the recovered signal originates in the same periodic mechanical displacement (77, 78). Figure 6 illustrates a representative sensor-based acquisition chain, in which the transduced signal is digitised and passed to a DSP stage for vital sign extraction, a structure common to the modalities discussed below.

**Figure 6:**
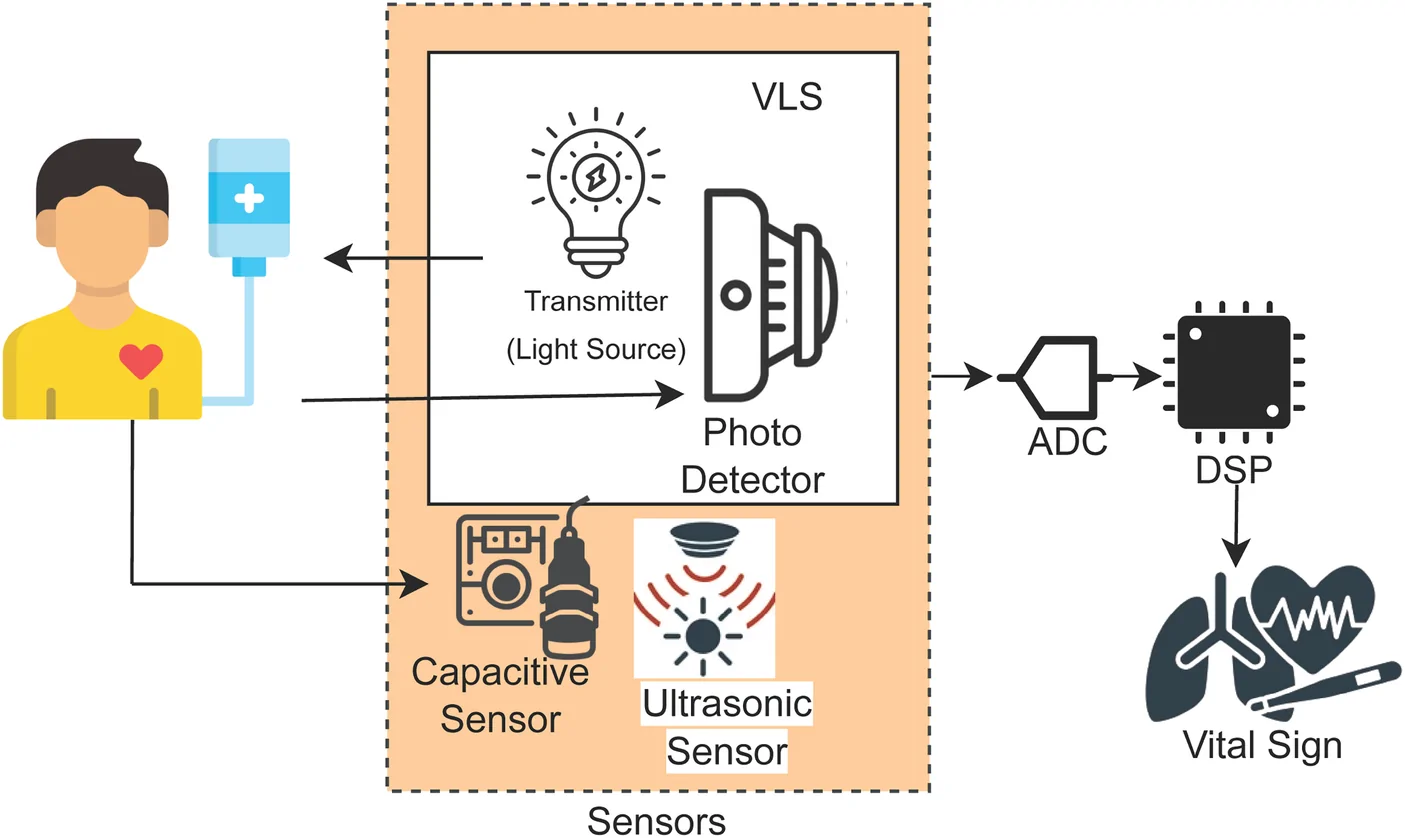
Sensor based system. Visible light sensing, capacitive, and ultrasonic transducers encode periodic chest displacement through different physical mechanisms, but share a common acquisition chain: the transduced signal is digitised and passed to a signal processing stage for vital sign extraction. (VLS: Visible Light Sensing, ADC: Analog-to-Digital Converter, DSP: Digital Signal Processor.)

The study (3), utilizes VLS which detects HR and RR by analyzing changes in the light reflected from a person’s body. This method uses a simple visible light source and photodetector, coupled with signal processing algorithms, to convert small variations in light reflection into precise vital sign measurements.

Using the same optical transmitter and photodetector arrangement, speckle vibrometry replaces intensity measurement with coherent speckle analysis. Illuminating the skin with coherent light produces a speckle pattern that shifts as the surface vibrates, and tracking this shift yields a mechanical signal from which HR is recovered (77). Because individual cardiac events are resolved rather than a dominant spectral peak, the method supports instantaneous HR estimation and hence beat-to-beat interval measurement, in contrast to approaches reporting only an averaged rate over a window. A comparable optical principle underlies fiber-based transduction, where displacement transmitted to an embedded fiber modulates the guided light; short-term HRV indices extracted this way have been mapped to an inferred fatigue state, indicating that unobtrusive optical sensing can support autonomic assessment rather than rate measurement alone (78).

Another method described in Tang et al. (76) employs highly sensitive passive EPS for non-contact detection of biophysical signals. These sensors incorporate an optimized TIA and a single guarded sensing electrode to improve sensitivity. The system also integrates an ACL to reduce power line interference and motion artifacts, ensuring reliable signal detection in noisy environments.

In Kusche et al. (75), ultrasonic and capacitive sensors are used to monitor vital signs without physical contact. Ultrasonic sensors detect tiny chest wall vibrations by measuring minute changes in distance, less than 2μm, resulting from thoracic movements during breathing and heartbeats. Capacitive sensors measure the distance between the sensor and the surface of the body, enabling effective RR monitoring even when the subject is clothed, by exploiting the electrical differences between human tissue, air, and clothing. Where the sensing element is placed in the surface supporting the subject rather than directed at the body, the recorded quantity becomes the mechanical recoil produced by cardiac ejection and respiration. Ballistocardiographic acquisition of this kind has been applied during acupuncture sessions, in which the subject remains still for an extended period, using a multi-task formulation that estimates several vital signs jointly from the single recorded waveform rather than processing each independently (79). Table 9 illustrates an overview of methods along with opportunities and challenges of sensor based non contact vital sign monitoring system.

**Table 9:** Opportunities and challenges of sensor-based vital sign monitoring systems.

Ref.	Database	Vital Sign	Methods	Quantitative Performance	Opportunities	Challenges
(3)	IEEE Xplore	HR, RR	VLS technology	Accuracy(%): 94 (HR, RR)	Low-cost ubiquitous health monitoring applications	Motion artifacts; lighting sensitivity
(76)	IEEE Xplore	HR and HRV through ECG, RR, EEG	EPS technology	–	Non-contact multimodal sensing with extended range	Position sensitivity; power-line interference
(75)	IEEE Xplore	HR and RR	Ultrasonic Sensors, Capacitive Sensors	–	Patient comfort; versatile applications	Environmental sensitivity; resolution limitations
(79)	PubMed	HR, RR	Non-contact under-mattress BCG (PVDF film)	HR MAE 0.77 bpm, RR MAE 0.43 brpm (best case); overall MAE 0.81, MAPE 2.53%, $R^{2}=99.77\%$	Cable-free; low computational load; real-time; cheaper and interference-tolerant vs. radar/fibre	Noise-sensitive; tilt- and placement-dependent; young male cohort; unvalidated under needling
(77)	PubMed	HR	Laser speckle vibrometry	–	Skin-exposure-free HR; relaxed aiming; lighting-independent; post-operative and burn use	Motion-sensitive; abnormal breathing and arrhythmias untested
(78)	PubMed	HR, RR, HRV (fatigue state)	FBG optical fibre sensing	–	Electrode-free BCG; EMI-immune; small and lightweight	Denoising required; baseline drift; motion artefacts

#### 3.1.6 Mixed system

Mixed system design in non-contact vital sign monitoring combines different sensing technologies, such as cameras and radars, to enhance the accuracy and reliability of detecting vital signs. Mixed camera–radar vital sign monitoring aims to estimate the underlying plethysmographic waveform

$$v(t)=a_{0}+a_{R}\sin(2\pi f_{R}t)+a_{H}\sin(2\pi f_{H}t)+\eta_{v}(t),$$ (16)

where $f_{R}$ and $f_{H}$ are the respiration and heart-rate frequencies, $a_{R}$ and $a_{H}$ are their amplitudes, and $\eta_{v}(t)$ aggregates physiological variability. After modality-specific preprocessing (rPPG extraction from the camera stream and phase-/range-domain processing from radar), the camera and radar measurements can be approximated as

$$y_{\text{cam}}(t)=\alpha_{\text{c}}\;v(t)+\beta_{\text{c}}+n_{\text{cam}}(t),$$ (17)

$$y_{\text{rad}}(t)=\alpha_{\text{r}}\;v(t)+\beta_{\text{r}}+n_{\text{rad}}(t),$$ (18)

where $\alpha_{\text{c}},\alpha_{\text{r}}$ are modality gains, $\beta_{\text{c}},\beta_{\text{r}}$ are offsets, and $n_{\text{cam}}(t),n_{\text{rad}}(t)$ denote modality-specific noise and artifacts (e.g., skin-tone dependent noise for camera and motion-/multipath-induced noise for radar). Stacking the two channels yields

$$y(t)=\left[\begin{array}{c} y_{\text{cam}}(t)\\ y_{\text{rad}}(t) \end{array}\right]=\left[\begin{array}{c} \alpha_{\text{c}}\\ \alpha_{\text{r}} \end{array}\right]v(t)+\left[\begin{array}{c} \beta_{\text{c}}\\ \beta_{\text{r}} \end{array}\right]+n(t),$$ (19)

with $n(t)=[n_{\text{cam}}(t)n_{\text{rad}}(t){]}^{T}$. A simple linear fusion model then estimates the plethysmographic waveform as

$$\hat{v}(t)=w^{T}y(t),$$ (20)

where w is a fusion weight vector (learned or designed to account for SNR and fairness), and heart and respiration rates are obtained from the spectrum of $\hat{v}(t)$ via peak detection. Figure 7 shows a representative mixed-system pipeline, in which vision-based landmark detection localises the region of interest and the radar channel then supplies the movement estimate from which vital signs are derived.

**Figure 7:**
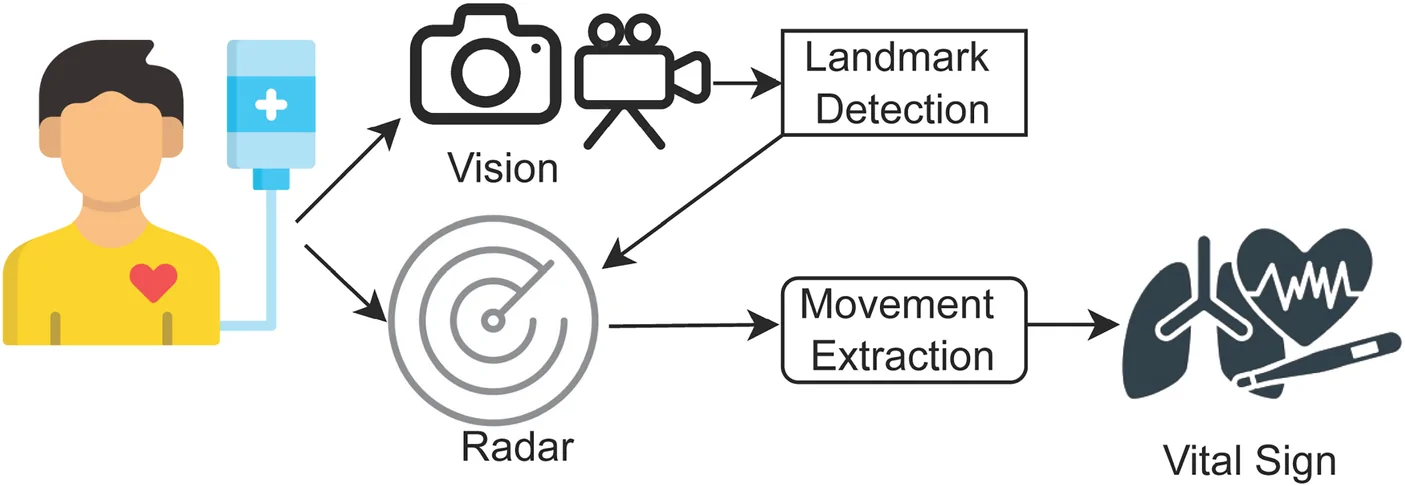
Mixed system, in which two or more sensing modalities are combined to estimate the same vital signs. In the pipeline shown, vision-based landmark detection localises the region of interest and guides the radar channel, from which movement extraction yields the vital signs.

In Shokouhmand et al. (80), an RGB-D camera is utilized to determine the subject’s position and provide coordinates, which are then used to guide an FMCW radar. The radar employs a beam-steering technique to focus on specific regions identified by the camera, enabling it to monitor heart and lung activities. Another study (81) introduces an innovative method to estimate plethysmograph signals by merging data from biased camera-based imaging (iPPG) and unbiased 77 GHz radar. This method seeks to improve HR monitoring by addressing skin tone biases in iPPG. The fusion approach combines the strengths of both technologies, improving signal quality and reducing bias compared to using a single modality.

Neonatal monitoring has become a recurring test case for mixed systems, since the subjects are small, frequently obscured by bedding or incubator surfaces, and cannot tolerate adhesive electrodes. Following the same vision-guided structure as Shokouhmand et al. (80), a radar–vision fusion system uses the visual channel to delineate anatomical regions and then separates thoracic from abdominal contributions to the radar-derived respiratory signal (82). This extends the role of the guidance stage beyond locating the subject: rather than aggregating chest-wall motion into a single displacement estimate, the segmentation allows spatially distinct components to be resolved, which matters clinically in neonates because divergence between thoracic and abdominal movement is itself a diagnostic indicator. Where the two modalities instead measure independently, as in Vilesov et al. (81), the combination occurs at the estimate level. Synchronous acquisition from a 3D time-of-flight camera and a microwave interferometric radar follows this pattern for neonatal RR, with the two channels evaluated jointly so that intervals in which one modality is unreliable can be identified from the other (83). Table 10 presents the studies that employ a mixed approach in non-contact vital sign monitoring systems.

**Table 10:** Opportunities and challenges of mixed system-based vital sign monitoring systems.

Ref.	Database	Vital Sign	Methods	Quantitative Performance	Opportunities	Challenges
(80)	IEEE Xplore	HR, RR	RGB-D camera with FMCW radar	Distance (0.5–2.5 m): Accuracy(%): 97.68 (RR) / 85.88 (HR), Azimuth (0${}^{\circ }$–30${}^{\circ }$): Accuracy(%): 90.02 (RR) / 86.05 (HR), Elevation (-30${}^{\circ }$–+30${}^{\circ }$): Accuracy(%): 96.71 (RR) / 89.50 (HR), Incident Angle (-30${}^{\circ }$–+30${}^{\circ }$): Accuracy(%): 97.52 (RR) / 86.71 (HR), Dual-Subject (20–70 cm): Accuracy(%): 92.26 (RR) / 73.23 (HR)	Real-time multimodal integration	Motion; clutter; distance limitations
(81)	ACM	HR, RR, BP	RGB camera and 77 GHz radar	MAE: 1.12, MAPE: 1.52, RMSE 3.42	Multimodal enhancement	Skin-tone variability
(83)	PubMed	RR (HR via radar)	ToF depth camera + CW microwave interferometric radar for neonates, evaluated synchronously	ToF error ≤3 bpm (20–60 bpm) but 11–31 bpm (5–10 bpm); radar ≤3 bpm with outliers; update rate 50 ms (radar) vs. ∼7.5 s (ToF)	No pressure marks or skin irritation; complementary sensor fusion	ToF noise- and reflection-sensitive, fails at low RR; radar motion- and multipath-sensitive
(82)	PubMed	HR, RR	MIMO mmWave radar (SFCW) and radar–vision fusion beam guidance;	RR MAE 1.51–3.35 bpm; HR MAE 3.01–4.12 bpm	Contact-free (no setup delay or skin damage); abdominal/thoracic separation for neonates; vision-guided beamforming robust to motion and staff	Staff-induced phase mutations; wrong-target locking; harmonic overlap; wide-beam filtering loss; camera dependency

### 3.2 RQ2: what are the opportunities associated with non-contact vital sign monitoring?

Non-contact vital sign monitoring offers several opportunities, especially in healthcare settings where patient comfort and safety are a priority. It eliminates the need for physical contact, making it ideal for sensitive populations, such as infants, the elderly, and individuals with compromised skin integrity. These systems enable continuous and remote monitoring, which is particularly valuable in telemedicine and home care, reducing the need for frequent hospital visits. To explore RQ2 based on several system designs described in 3.1, we identified 4 main opportunities. These opportunities include streamlined health monitoring, essential traits of smart monitoring, efficiency, and diverse applications as depicted in the right side of Figure 8.

**Figure 8:**
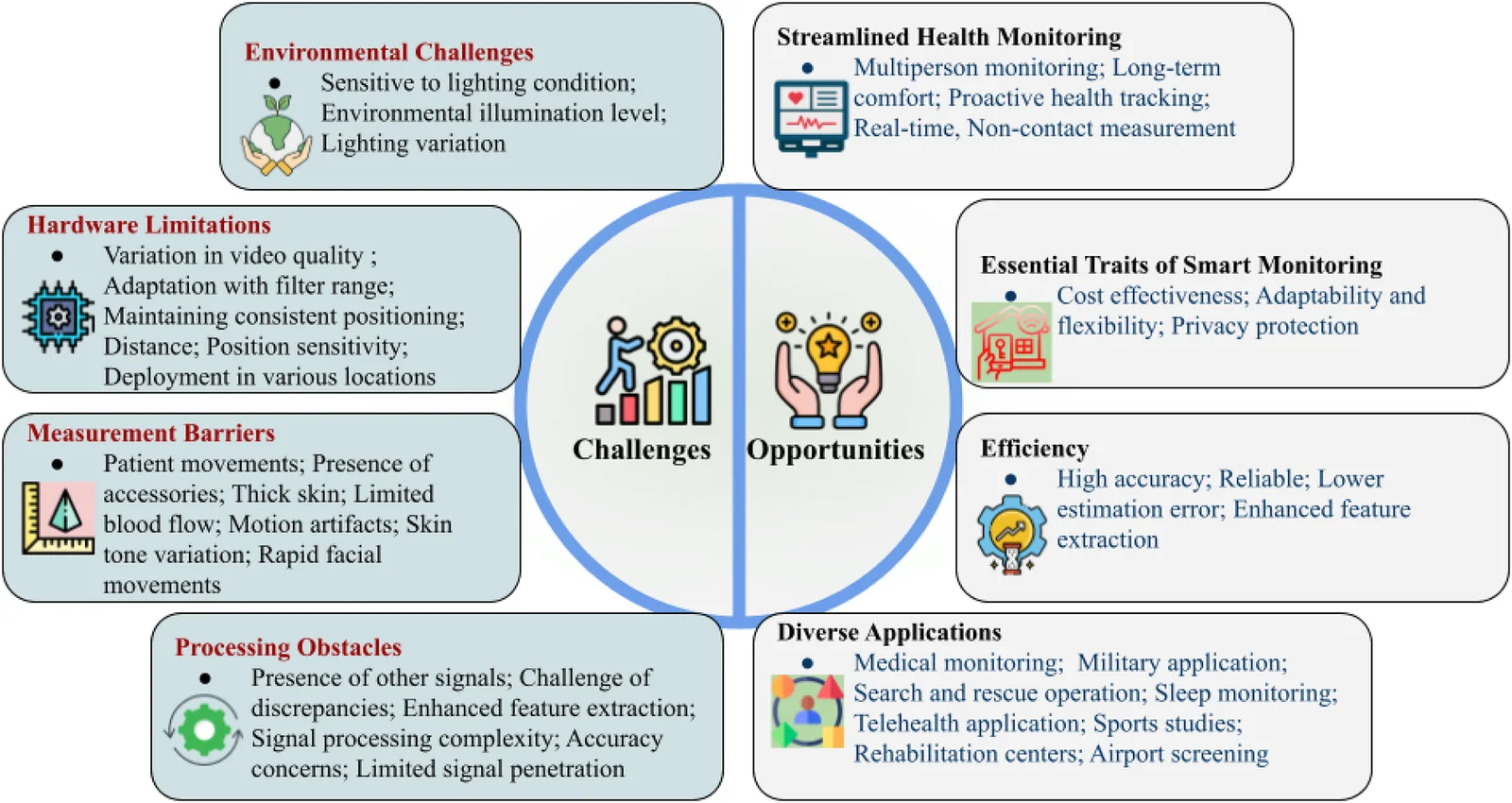
Challenges vs. opportunities of non-contact vital sign monitoring.

#### 3.2.1 Streamlined health monitoring

Streamlined health monitoring refers to a simplified, efficient approach to healthcare that enhances both the quality of care and the user experience by focusing on key features that make vital sign monitoring seamless and accessible. This approach integrates several critical elements:

- **Multiperson monitoring:** This feature enables the simultaneous tracking of multiple individuals’ health data in real-time. It supports the monitoring of large groups, such as families, communities, or patients in healthcare settings. By streamlining the collection and analysis of health data from multiple individuals, healthcare providers can easily identify trends and offer timely interventions for each person. Authors such as Eder and Eldar (11), Hu et al. (9), Petrović et al. (50), Wang et al. (46), Wu et al. (88), Wang et al. (7), Soto et al. (99), Li et al. (60), and Guan et al. (59) have implemented this approach. More recent MIMO-based work extends this to targets that are closely spaced or partially occluded, with blind target counting removing the need to specify the number of subjects in advance (32, 42), and dual-antenna designs maintaining subject identity across movement (97).
- **Comfortable and long-term monitoring:** Because nothing is attached to the body, non-contact systems can operate continuously over extended periods without compromising the patient’s comfort or interfering with daily routine. Advanced sensing and signal processing allow accurate measurement at a distance, so reliable data collection over long durations is achieved without the discomfort of worn devices (10). Vision-based methods using RGB cameras avoid skin irritation and allergic reaction associated with adhesive electrodes and wearable straps during prolonged use (70). Sensor-based research, such as the work presented in Kusche et al. (75), also emphasizes improving patient comfort. Unobtrusiveness has since been demonstrated over clinically realistic durations: thermal cameras have recorded respiration across full overnight sleep studies without disturbing the subject (62), under-mattress ballistocardiography operates cable-free beneath a stationary subject (79), and IR-UWB sensing has been sustained over 12-hr daily-life recordings (47).
- **Proactive health monitoring:** Streamlined health monitoring emphasizes early detection and prevention rather than reactive interventions. By continuously collecting data, these systems can alert users and healthcare providers to potential health issues before they become critical. This proactive approach encourages early treatment, reducing the need for emergency care and improving long-term health outcomes. The paper (6) emphasizes the need to shift from reactive disease treatment to proactive health monitoring. This approach involves regular monitoring of vital signs to detect potential health issues early, allowing for timely intervention and prevention of adverse health outcomes. This early-detection role now has clinical evidence: radar-based acquisition has been evaluated for triage across 315 emergency department patients (44), ward-deployed radar has been used for deterioration alerting (98), and acoustic sensing has been applied to screen for occupational respiratory disease (57).
- **Non-contact and real-time monitoring:** Non-contact technologies, such as sensors and infrared cameras, enable continuous monitoring without physical interference. Real-time data collection ensures that healthcare providers receive up-to-the-minute updates on a patient’s condition. For instance, HR, RR, and SpO${}_{2}$ levels can be continuously monitored without direct contact, reducing the risk of infection while ensuring privacy and comfort for the patient. The proposed method in Dai et al. (16) operates in a quasi-realtime manner, capable of processing raw data and displaying estimated RR and HR in less than one second. This rapid processing capability ensures that vital sign information is updated continuously, providing real-time insights into the subject’s health status. The system presented in Petrović et al. (50) is designed to be computationally efficient, delivering real-time performance even on platforms with limited hardware resources. This efficiency minimizes the total HR detection delay, which is critical for applications requiring fast BBI detection. Additionally, the method described in Shokouhmand et al. (80) processes data in real-time by segmenting RR and HR signals into 30-sec overlapping windows. This approach supports continuous monitoring and provides timely updates of vital sign measurements. Edge-resident implementations now report explicit timing: thermal RR estimation runs at 22.5 FPS on a Jetson Orin Nano (61), and gridless spectral estimation reduces per-estimate cost from 32.74 s to 0.06 s, making real-time IoMT deployment feasible (38).

Together, these elements of streamlined health monitoring provide a more efficient and user-friendly approach to healthcare. This comprehensive framework enhances the quality of care while reducing the burden on both patients and healthcare providers.

#### 3.2.2 Essential traits of smart monitoring

- **Cost effectiveness:**
  Contactless vital sign monitoring systems ensure accessibility across various settings such as hospitals, home care, or low-resource environments. Abuella and Ekin (3), Guan et al. (59), Ambrosanio et al. (53), Ling et al. (34), Al Ahmad and Olule (6) highlighted cost-effectiveness in their infrastructure. By leveraging low-cost sensors and scalable software solutions, these systems reduce deployment and operational costs. Their minimal infrastructure requirements enable a high return on investment compared to traditional monitoring setups. Recent work reinforces this by targeting commodity hardware explicitly: RR estimation on consumer-grade Wi-Fi chipsets rather than research CSI platforms (58), and BP estimation from ordinary RGB cameras (73).
- **Adaptability and flexibility:**
  Some contactless vital sign monitoring systems adapt to diverse scenarios, such as varying light conditions, different skin tones, and dynamic user movements. Flexibility in integrating with other healthcare technologies or workflows allows the system to support a wide range of patient needs. This adaptability enables deployment across diverse environments, from ICU settings to outpatient monitoring at home. For example, radar technology is scalable and can be adapted for use in diverse environments, from hospital settings to personal health monitoring systems (86). The use of commodity Wi-Fi devices enables the system to resolve individuals in crowded environments and monitor multiple people at once (60). EVM with RGB video has shown strong performance in NICU environments, even under varied lighting conditions (67). EVM works effectively under different lighting and with a variety of skin types, offering better monitoring capabilities (13). RGB and thermal imaging systems also provide robust solutions for monitoring under diverse conditions, including motion and varying breathing rates (65). Depth and thermal sensing extend adaptability further, since neither depends on skin tone or ambient illumination: depth-based RR has been validated in an anaesthesia setting with obstructed sightlines (64), and ToF sensing has held from 5 to 1,800 lux in a moving vehicle (108).
- **Privacy protection:**
  Since contactless monitoring often involves capturing images, videos, or other sensitive biometric data, robust privacy mechanisms are critical. As radar-based systems do not require physical contact with the patient, they inherently offer higher levels of privacy compared to traditional monitoring methods (10). Region-resolved radar imaging strengthens this further, since the reconstructed representation carries no identifying visual detail (42), and edge-resident thermal processing avoids transmitting any imagery off-device (61).

#### 3.2.3 Efficiency

Contactless systems ensure accurate and consistent readings of vital signs, such as HR, RR, and SpO${}_{2}$, regardless of environmental or user-specific variations. This reliability enhances the system’s trustworthiness and usability in critical healthcare scenarios. For instance, radar-based systems, particularly FMCW radar, provide real-time monitoring capabilities with high accuracy, making them suitable for dynamic environments where patients may move or change position (9). Furthermore, the method presented in Petrović et al. (50) demonstrates reliability and accuracy through validation on a group of ten subjects. The study shows a high level of agreement between BBIs extracted from doppler radar signals and reference ECG signals. With a low mean relative error for BBI estimation, this method demonstrates strong potential for precise and reliable real-time monitoring. Another study (9) reports high measurement accuracy even when subjects are moving at speeds of up to 1 m/s. In this scenario, the RMSE remains below 4.05 bpm. The method also performs well along random movement routes, achieving RMSE values below 6.85 bpm. Using RGB cameras and 77 GHz radar, together with additional sensing modalities such as thermal and acoustic sensors, enhances the quality and depth of the collected datasets (81). The use of advanced signal processing techniques (6), including FFT, enables precise extraction of vital sign features from radar signals, ensuring consistency with reference measurements and supporting reliable continuous monitoring. Additionally, operating at higher frequencies, such as 122 GHz, improves resolution and increases immunity to interference, further enhancing system accuracy and reliability. These advantages make non-contact monitoring systems a promising tool for enhancing patient care and enabling continuous, non-invasive health monitoring.

#### 3.2.4 Diverse application

Non-contact vital sign monitoring leverages advanced technologies such as radar, infrared, sound, vision, and optical sensors to measure physiological parameters without physical contact, offering numerous advantages across diverse fields. In medical monitoring, radar systems enable simultaneous tracking of multiple individuals, making them well suited for crowded environments such as hospitals and clinics (11). In contrast, VLS offers a low-cost, safe, and widely available solution, with applications spanning health monitoring, advanced medical facilities, gaming, and virtual reality (3). Ultrasonic and capacitive sensors enhance patient comfort and are widely used in fitness, automotive industries, and heart activity monitoring (75). Radar technology is highly scalable and supports deployment across diverse environments, ranging from hospital settings to personal health monitoring systems (86). It is also valuable in high-risk or remote scenarios, such as search and rescue operations, where contact-free monitoring is essential (35). Furthermore, technologies such as EVM and rPPG enable safer, contact-free measurement of vital signs, reducing infection risks and supporting telehealth applications (69). These systems are also applied in sports studies, rehabilitation centers, quarantine facilities, hospital and airport screenings, and automotive industries, highlighting their versatility across a wide range of domains (12). Newer deployments extend the range further: neonatal intensive care (82, 83, 109), in-vehicle driver monitoring (48, 108), CT suites (63), and post-anaesthesia recovery (64).

### 3.3 RQ3: what are the challenges associated with non-contact vital sign monitoring?

Non-contact vital sign monitoring has garnered significant attention due to its broad applications in healthcare, wellness, and remote monitoring. However, its effectiveness and reliability depend on overcoming several challenges. Across the various system designs analyzed in the 88 studies, these challenges can be generalized into four main categories, as illustrated in left side of Figure 8. This categorization provides a structured view of the key obstacles faced, helping to identify specific areas requiring targeted solutions for advancing non-contact vital sign monitoring technologies.

#### 3.3.1 Environmental challenges

Non-contact vital sign monitoring faces several challenges related to environmental conditions, lighting variations, and signal interference, all of which can significantly affect accuracy and reliability. Technologies such as VLS and rPPG are highly sensitive to lighting variations. Changes in ambient illumination, including shifts between natural and artificial light, shadows, or flickering, can introduce noise and reduce signal quality. Low-light environments often result in weak or unstable signal acquisition. Conversely, excessive brightness or glare can obscure vital sign measurements and degrade accuracy. Video-based methods, including EVM and RGB video, face challenges from varying lighting, discrepancies in RR estimates, and interference caused by skin characteristics, accessories like glasses or hairstyles, and motion artifacts (12, 67, 69). Multispectral video and RGB-D camera systems with FMCW radar encounter additional difficulties due to environmental factors like temperature, humidity, and distance limitations, as well as interference from involuntary movements (68, 80). Furthermore, Wi-Fi signal attenuation and environmental noise can degrade radar performance over distance, while physical barriers such as walls or furniture pose additional obstacles (7, 95). Addressing these challenges through robust algorithms and adaptive techniques is essential to ensure reliable performance. Such improvements are also critical for the widespread adoption of non-contact monitoring systems in real-world applications. Recent work quantifies these effects more precisely. Radar performance degrades with angular offset beyond $30^{\circ }$ and at low SNR (38), and nearby metal surfaces raise HR error from under 4 bpm to approximately 14 bpm (37). Thermal imaging depends on the contrast between exhaled air and ambient temperature, so accuracy falls in warm environments (61), while depth-based measurement in clinical settings is confounded by warming blankets and obstructed sightlines (64). Reported ranges remain short across modalities, typically 0.2–2 m for radar (40) and under 2 m for thermal sensing (61).

#### 3.3.2 Hardware limitations

Contact-based vital sign monitoring systems, such as those using sensors and wearables, face significant challenges due to hardware limitations. These limitations can affect the accuracy, reliability, and usability of the devices in real-world applications. For instance, motion artifacts caused by user movement can introduce noise and distort the signals detected by contact sensors. Mitigating these effects often requires advanced filtering techniques and robust hardware design (3). Additionally, the sensitivity of sensors to environmental factors such as temperature and humidity can lead to inconsistent performance, particularly in non-ideal conditions (75).

Another major challenge lies in the limited signal penetration of certain technologies like VLS and ultrasonic sensors, which can struggle to provide accurate readings in cases where the signal path is obstructed or degraded (3, 75). Deployment difficulties also arise when hardware needs to be adapted to diverse locations or physiological characteristics, further complicating their practical use. For example, capacitive sensors are highly sensitive to the user’s position and require precise alignment to function optimally, which is not always feasible in dynamic environments (76).

Moreover, PLI is a recurring issue in contact-based systems that rely on electrical signals. This interference often requires additional shielding or noise-cancellation techniques to maintain signal integrity (76). The technical limitations of hardware components, together with their susceptibility to external disturbances, underscore the need for continued innovation. Improving sensor robustness, resolution, and adaptability remains essential for advancing contact-based vital sign monitoring systems. Hardware constraints also arise at the system level rather than in the transducer alone. Cuff-less BP estimation has required two synchronised radars and per-subject calibration (43), and Doppler implementations remain bulky and sensitive to ambient vibration (49). Data volume is a further constraint: region-resolved MIMO imaging generates roughly 100 GB per hour, which precludes real-time operation on constrained hardware (42).

#### 3.3.3 Processing obstacles

Non-contact vital sign monitoring systems face various technical challenges that affect their performance, accuracy, and reliability. One major issue is the presence of other signals, which can interfere with the accurate detection of vital signs. For instance, respiratory harmonics or overlapping physiological signals, such as breathing and heartbeat, complicate the separation and extraction of individual vital sign measurements (10, 53).

The challenge of discrepancies in measurements, particularly when different methods or algorithms are employed, further exacerbates the problem. Techniques like EVM often face discrepancies in RR estimates due to varying environmental conditions and differences in signal acquisition techniques (67).

Additionally, enhanced feature extraction becomes critical in environments with complex and dynamic conditions. Non-contact systems often need to extract subtle features from noisy or weak signals, which requires advanced algorithms and hardware capable of high precision (13). This challenge is further compounded by the complexity of signal processing. Vision-based and radar systems must handle large volumes of data in real time to mitigate motion artifacts, lighting variations, and environmental noise (102).

Accuracy concerns remain a pervasive challenge, as non-contact systems can be affected by numerous external factors such as lighting conditions, motion artifacts, and subject variability. For example, video-based methods are sensitive to skin characteristics, head motion, and ambient lighting, which can significantly impact the accuracy of HR and respiratory measurements (12).

The limited signal penetration of non-contact systems, especially those based on visible light or thermal imaging, restricts their ability to capture vital signs in the presence of obstructions, and also affects scenarios where signals must penetrate thick tissue. This limitation often requires optimal positioning and careful environmental control to ensure reliable data acquisition (3, 75).

Recent studies quantify several of these obstacles. Harmonic interference remains the dominant one: respiratory harmonics overlap the cardiac band and are suppressed using empirically set bounds rather than analytically derived ones (40), and the same interference corrupts pulse oximetry estimates recovered from camera data (109). Irregular breathing degrades RR accuracy by nearly 20% (40), and video compression weakens the rPPG signal enough to introduce harmonic rate errors (72). Computational cost is a further barrier where optimisation-based decomposition is used, since the search stage can exceed real-time budgets (37), although gridless spectral estimation has reduced per-estimate cost from 32.74 s to 0.06 s (38).

These challenges highlight the need for continuous innovation in algorithm development, hardware design, and system integration to improve the accuracy and robustness of non-contact vital sign monitoring in diverse settings.

#### 3.3.4 Measurement barriers

Non-contact vital sign monitoring systems face significant challenges when dealing with various physical and environmental factors that impede accurate measurements. One such challenge is patient movements, which introduce noise and distort the captured signals. Movements such as posture changes or dynamic body positioning can severely impact the precision of monitoring systems, especially those reliant on radar or video-based methods (96).

The presence of accessories, such as eyeglasses, jewelry, or certain hairstyles, further complicates measurement accuracy. These items can obscure regions of interest or reflect light in unintended ways, interfering with vPPG or thermal imaging techniques (69). Thick skin and limited blood flow in certain regions of the body present additional challenges, particularly for video-based systems that rely on subtle color changes caused by blood perfusion. Regions with low vascularization, such as the torso or limbs, often yield weaker signals, making vital sign extraction more difficult (13).

Motion artifacts are another pervasive barrier. Rapid movements of the head or other body parts can distort signals and hinder the ability of algorithms to accurately identify and process vital sign data. This issue is particularly prominent in real-world applications where patients may not remain stationary (102). Moreover, skin tone variation significantly affects the performance of optical and video-based methods. Darker skin tones absorb more light and reflect less, which reduces the signal-to-noise ratio for techniques that rely on reflected light to detect subtle physiological changes (81).

Lastly, rapid facial movements create challenges for ROI detection and tracking. Non-contact systems, especially those using RGB cameras, struggle to maintain accurate monitoring when individuals exhibit rapid expressions or facial gestures, leading to errors in measurement (65).

Several studies quantify how narrow the operating conditions remain. Radar accuracy is posture-dependent, with RR error rising from 0.06 to 0.80 brpm between prone and lateral positions (42), and multi-target performance degrades under occlusion and close angular spacing (32). Emergency-department deployment required 150 s of stillness and discarded 30% of recordings (44). Clothing attenuates both depth (63) and ToF signals, raising RR error to 3.57 bpm under multiple layers (108). These figures indicate that the barriers described above are not merely qualitative: they define the conditions under which reported performance holds.

These barriers underline the need for inclusive, adaptive algorithms and robust hardware capable of mitigating the effects of such physical and environmental factors in non-contact vital sign monitoring.

## 4 Discussion

The systematic review aims to address three key research questions concerning the system designs, opportunities, and challenges of non-contact vital sign monitoring.

### 4.1 Distributions of system architectures and vital signs

Tables 5–10 present an overview of the 88 studies included in the review, detailing the various system designs, opportunities, and challenges associated with contactless vital sign monitoring. Additionally, Figure 9 illustrates the system-wise distribution of the primary studies selected from various journals, providing a comprehensive visualization of the research landscape in this domain.

**Figure 9:**
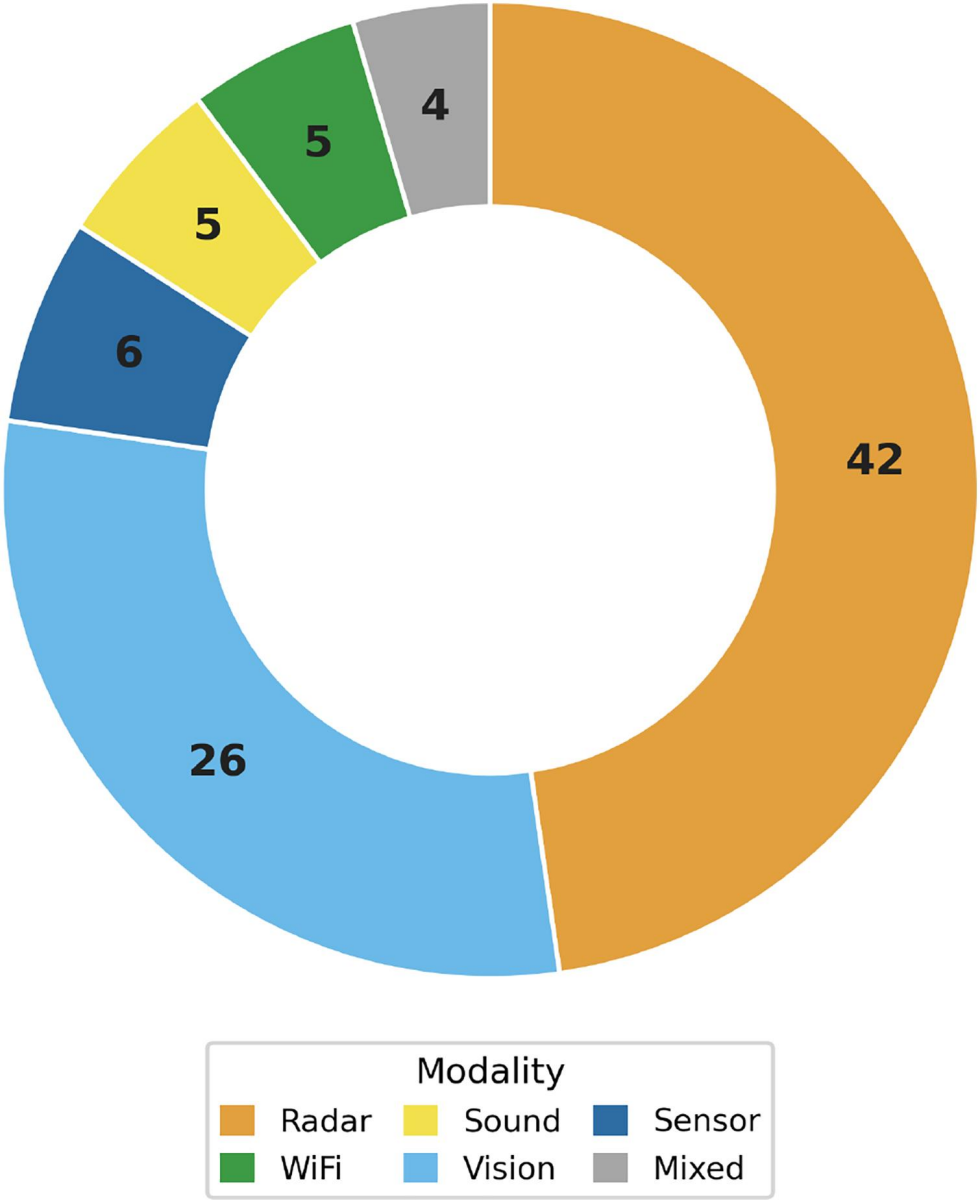
Distribution based on system design of non-contact vital sign monitoring.

The distribution of architectural approaches reveals distinct trends in the field. As shown in Figure 9, the distribution of architectural approaches used in non-contact vital sign monitoring, based on 88 studies, is as follows. **Radar** is the most commonly used base system, with 42 out of 88 studies (approximately 47.7%) employing radar-based approaches. Radar-based systems are favored due to their high accuracy, long-range capabilities, and ability to monitor multiple vital signs without physical contact. **Vision-based** systems come next, with 26 out of 88 studies representing approximately 29.5%. These approaches leverage optical sensors to monitor vital signs. However, their performance may be affected by lighting conditions. **Wi-Fi-based** systems account for 5 out of 88 studies (approximately 5.7%), utilizing Wi-Fi signals for motion and respiration tracking and benefiting from existing communication infrastructure. **Sensor-based** systems represent 6 out of 88 studies (approximately 6.8%) and are often integrated with other technologies to enhance monitoring accuracy. **Mixed system designs**, which combine radar, vision, or sensor technologies, make up 4 out of 88 studies (approximately 4.5%), indicating a growing interest in hybrid solutions. Finally, **sound-based** systems, though still emerging, account for 5 out of 88 studies (approximately 5.7%), showing promise for specific non-contact monitoring applications. These findings highlight radar and vision as the dominant approaches, which together account for roughly three-quarters of the corpus, while the remaining four modalities are each represented by fewer than ten studies. This concentration suggests that the field has consolidated around two mature sensing paradigms, and that sound, Wi-Fi, sensor, and mixed designs remain at a comparatively early stage of validation rather than being established alternatives.

The distribution based on research work that used vital signs is shown in Figure 10. The distribution of vital signs used across the 88 reviewed studies is as follows. RR appears in the highest number of studies, being used in 73 out of 88 studies. This highlights its prominent role in non-contact vital sign monitoring, as it is crucial for tracking respiratory health, particularly for conditions such as apnea and hypo-ventilation. HR is also frequently used, included in 59 studies, reflecting its importance as a crucial indicator of cardiovascular health. BP is utilized in 10 studies, which suggests its relevance in cardiovascular monitoring, although it is less commonly employed than RR and HR. HRV is used in 10 studies as well, reflecting a growing interest in monitoring autonomic nervous system activity and stress levels. IBI is included in only 1 study, indicating a more niche application in heart health monitoring. Temperature is used in 3 studies, underlining its role in detecting fever and infections. Pulse rate appears in 2 studies, suggesting it is less commonly employed in non-contact monitoring scenarios. Finally, SpO${}_{2}$ is included in 5 studies, pointing to its relevance in monitoring SpO${}_{2}$ levels, which are crucial for assessing respiratory and cardiovascular conditions. Because most systems report more than one parameter, these counts sum to more than the number of included studies. This distribution emphasizes the dominance of RR and HR in non-contact vital sign monitoring, while other vital signs such as BP, HRV, IBI, temperature, pulse rate, and SpO${}_{2}$ are used less frequently but are still important in specific applications. The gap is substantial: RR and HR appear in 83.0% and 67.0% of studies respectively, whereas no other parameter exceeds 11.4%. Both are recoverable from periodic chest-wall displacement or skin-colour variation, which every modality surveyed here measures directly. BP, SpO${}_{2}$, and temperature instead require pulse-waveform morphology, wavelength-specific absorption, or radiometric calibration, and their scarcity reflects that they must be inferred indirectly rather than read from a dominant periodic component.

**Figure 10:**
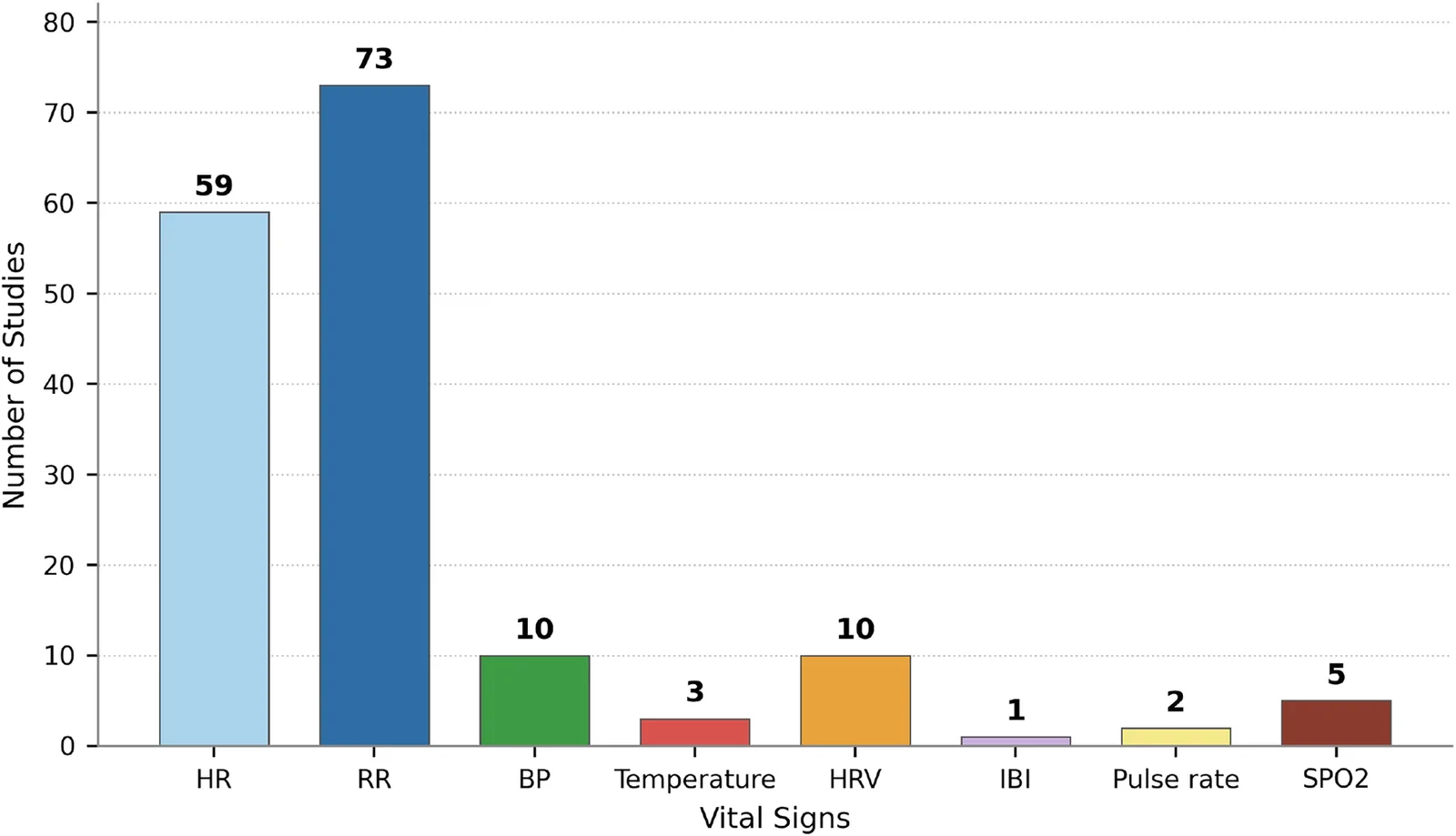
Distribution of research based on types of vital signs.

### 4.2 Architecture and performance comparison

As summarised in Tables 5–10, radar-based systems actively detect chest micro-motions and provide strong reliability even in low-light or partially occluded conditions, though their feasibility depends on advanced motion and clutter suppression. Vision-based systems, using RGB, depth, thermal, or multispectral imaging, offer low-cost and accessible options but are highly sensitive to lighting, occlusion, and motion artefacts. Sound-based systems can achieve very low errors using simple hardware, yet noise sensitivity and short operational range reduce their broader practicality. Wi-Fi-based systems leverage CSI variations from existing infrastructure to support multi-person sensing, but environmental dynamics introduce variability. Sensor-based systems (VLS, EPS, capacitive, ultrasonic) are easy to embed in physical environments, though their accuracy depends heavily on stable positioning. Mixed radar–vision architectures combine complementary modalities to enhance robustness but incur higher hardware and calibration complexity.

Quantitative comparison across modalities is constrained by reporting heterogeneity. Figures 11 and 12 therefore apply a fixed extraction rule: reported accuracy is taken only where expressed as a percentage, and error only where expressed as MAE, RMSE, or absolute error in BPM or BrPM, with signed bias and mean-difference values excluded as measures of agreement rather than error magnitude. Minima and maxima span across the studies within each modality, not within a single system. Applying this rule, no vision- or sound-based study in this review reports a percentage accuracy for HR or RR, using MAE, RMSE, or correlation instead, so those modalities are absent from Figure 11 rather than absent from the literature. The comparison below therefore reflects what each modality chooses to report as much as how it performs, and the two figures should be read together.

**Figure 11:**
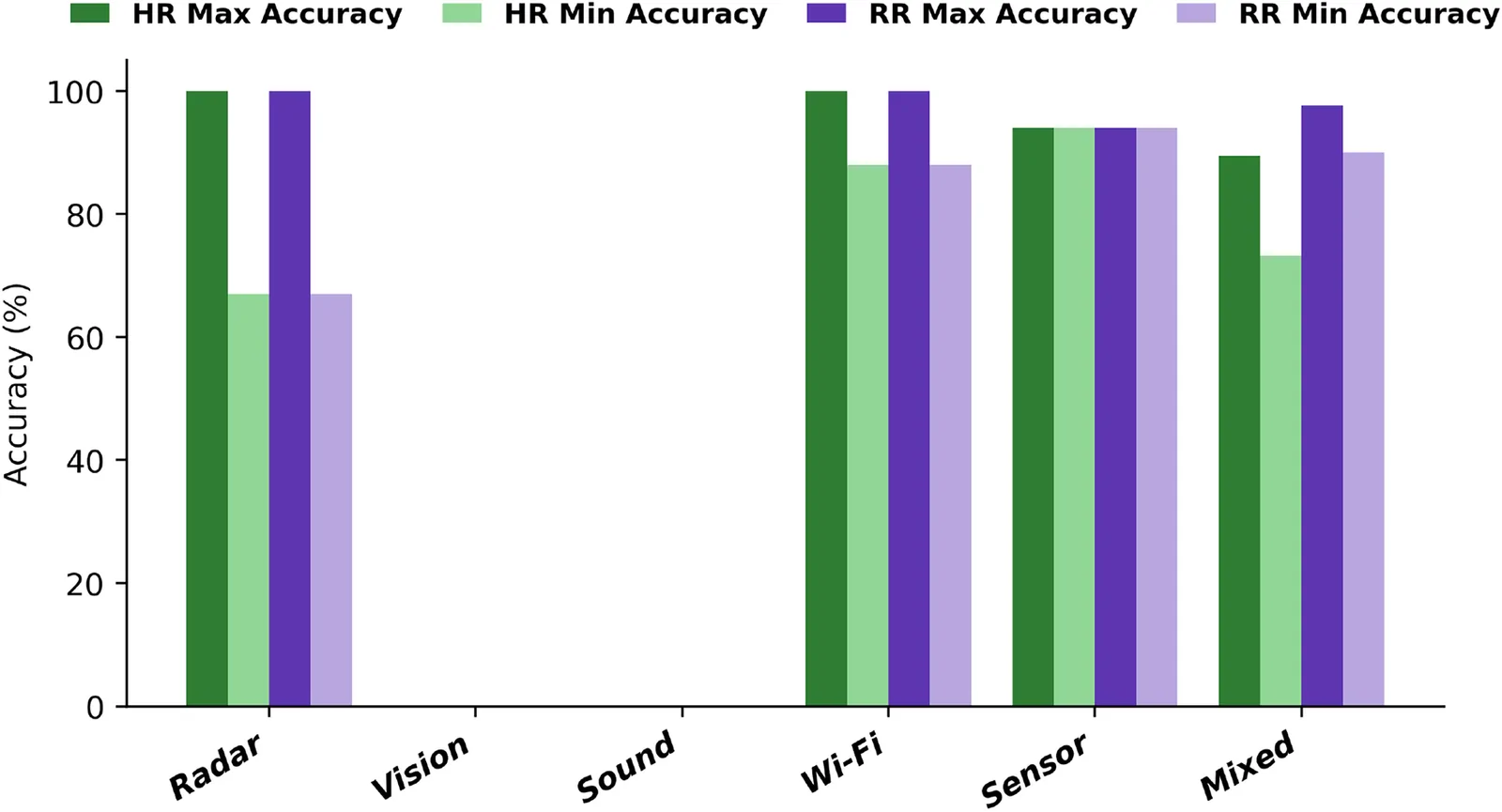
Maximum and minimum HR and RR accuracy across different modalities.

**Figure 12:**
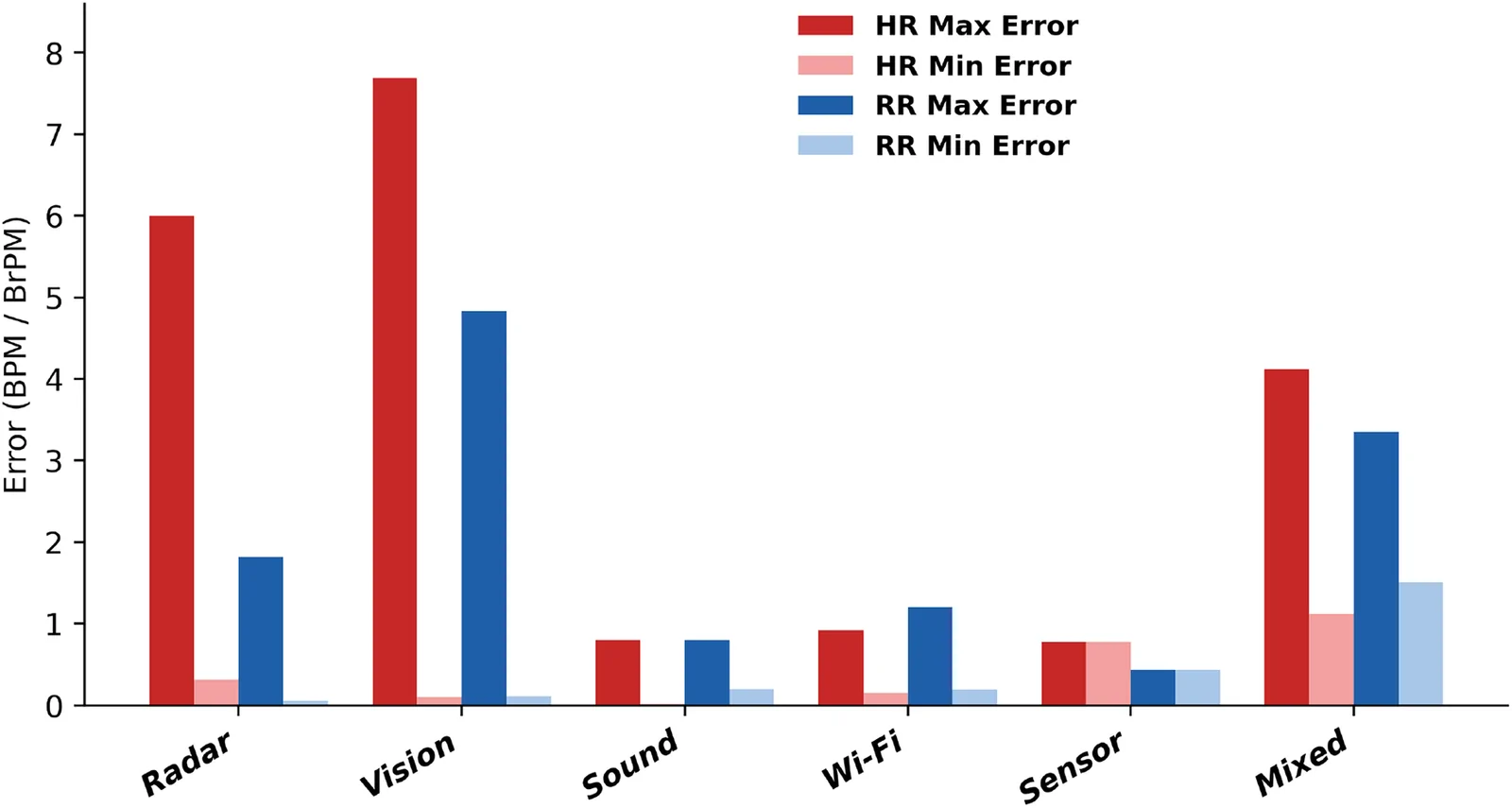
Maximum and minimum HR and RR error across different modalities.

Radar-based systems achieve the highest reported accuracy for HR and RR, reaching up to 100%, with sensor-based designs at 94% and mixed radar–vision systems reaching 97.7% for RR and 89.5% for HR. Wi-Fi systems demonstrate consistently strong performance, with HR accuracy near 97.9% and RR accuracy near 99.1%. Radar also shows the widest accuracy range (67–100%), reflecting the breadth of conditions under which it has been evaluated, while Wi-Fi exhibits a narrower range (88–100%). These comparisons rest on very unequal evidence: radar and vision contribute 42 and 26 studies respectively, whereas Wi-Fi, sound, sensor, and mixed designs contribute five, five, six, and four. Values for the latter four characterise individual systems rather than modalities, and we therefore make no statistical comparison between modalities.

Error distributions shown in Figure 12 indicate that sound-based systems achieve the lowest HR error (0.02 BPM) under controlled conditions , closely followed by vision at 0.10 BPM, though the sound figure derives from a single 40 kHz ultrasound study and is not representative of the modality as a whole. Radar attains the lowest RR error in the review (0.06 BrPM), and sensor-based designs, for which error data became available only with recent under-mattress BCG work, report 0.77 BPM for HR and 0.43 BrPM for RR. At the upper end, vision shows the largest errors of any modality (7.69 BPM for HR, 4.83 BrPM for RR), reflecting the difficulty of ventilated, low-light, and unconstrained-motion settings, with radar next at 6.00 BPM for HR. Sound and Wi-Fi span the narrowest error ranges (0.02–0.80 and 0.15–1.20 respectively), but this reflects their consistently controlled evaluation conditions — single stationary subjects at short range—rather than demonstrated robustness. Radar and vision span the widest ranges precisely because they have been evaluated across the most demanding scenarios, including moving subjects, multi-target scenes, occlusion, and clinical environments. Overall, radar offers the broadest validated operating envelope and the strongest RR precision, Wi-Fi and sound achieve tight errors within narrow tested conditions, vision provides the most accessible hardware at the cost of the widest error spread, and mixed systems trade higher calibration complexity for balanced performance across both parameters (1.12–4.12 BPM for HR, 1.51–3.35 BrPM for RR).

### 4.3 Clinical suitability of modalities

The clinical suitability of each modality can be understood by relating their architectural characteristics and performance patterns summarised in Tables 5–10. From a healthcare usability perspective, different non-contact sensing modalities align with distinct clinical workflows and patient care environments. Radar and Wi-Fi architectures are suitable for hospital wards and ICUs where continuous, hands-free monitoring is required and patients may be partially covered or unable to remain still. Vision- and sound-based systems are better suited for structured clinical spaces such as outpatient clinics or teleconsultation rooms, where environmental conditions can be kept stable to support reliable measurements. Sensor-based modalities are well matched to eldercare homes and long-term hospital beds, where unobtrusive integration into mattresses, chairs, or room infrastructure enables passive and sustained monitoring without caregiver intervention. Mixed radar–vision systems are most appropriate for specialised environments such as postoperative recovery or neonatal monitoring, where enhanced precision and robustness are essential. Overall, clinical suitability varies across modalities, and appropriate selection depends on workflow requirements, patient mobility, monitoring duration, and the precision needed for clinical decision support.

### 4.4 Opportunities and challenges across system designs

According to this systematic review and information presented in Table 5–10, non-contact-based vital sign monitoring technologies, including radar, vision, sound, sensor, Wi-Fi, and mixed system design, present unique opportunities and challenges in healthcare. Radar systems, such as IR-UWB and FMCW, enable non-contact monitoring to reduce infection transmission risk, particularly in clinical settings (11, 96). These systems can penetrate materials like clothing and curtains, allowing continuous monitoring without requiring a direct line of sight (96). However, radar systems are susceptible to accuracy degradation due to random body movements and posture variations, necessitating advanced signal processing techniques (88, 96). While FMCW radars are less affected by lighting and weather conditions, their low spatial resolution limits detailed monitoring compared to vision-based systems (11). Vision-based systems, such as RGB-D cameras, offer high spatial resolution, beneficial for detecting subtle physiological changes, and can be integrated with radar for enhanced accuracy through sensor fusion (80). Nonetheless, vision-based systems can be hindered by environmental lighting changes, clothing obstructions, and adverse weather, impacting their reliability (11, 80). Sound-based methods leverage acoustic signals for non-invasive monitoring and are typically more cost-effective than radar systems, which rely on specialized and expensive equipment (60). Additionally, sound-based systems do not require line-of-sight conditions, unlike vision-based methods that depend on clear visual access and can be affected by lighting and privacy concerns (60). Furthermore, sound-based monitoring avoids visual data capture, enhancing privacy in applications (60). However, environmental noise can limit their utility in some settings (60). Mixed system-based approaches combine technologies like radar and vision, enhancing robustness and adaptability across various environments by leveraging the strengths of each modality (80). Despite their potential, these systems face increased complexity and cost, which may restrict widespread adoption (80). Overall, the integration of multiple technologies holds significant promise for advancing the accuracy and applicability of non-contact vital sign monitoring in clinical and everyday environments.

## 5 Mitigation strategies

Non-contact-based vital sign monitoring systems, including radar, vision, sound, sensor, and mixed system design, are revolutionizing healthcare by providing noninvasive and continuous monitoring. Despite their potential, none of the studies in this survey addressed mitigation strategies for the challenges identified, nor did they explore the ethical and privacy concerns associated with these systems.

### 5.1 Measurement barriers

There are several approaches that could be incorporated to handle measurement barriers such as motion artifacts, patients’ movements, inter-patient variability, and many more. For instance, ANF with an Infinite Impulse Response-based design (110) effectively mitigates motion artifacts caused by body movements. Utilizing an adaptive algorithm, specifically the NLMS, the ANF is robust against both white and colored noise disturbances. This adaptability is crucial for handling sudden, time-varying movements, such as posture changes, even under challenging conditions. Spectral Matrix Decomposition-Based Motion Artifacts Removal (111) is another approach. The core of the method lies in recognizing that the spectral peaks of PPG signals and acceleration signals due to motion artifacts align in the frequency domain. Leveraging this observation, the process begins by framing the removal of motion artifacts as a spectral matrix decomposition optimization problem. To address the issue of skin tone variations, advanced signal processing techniques such as parameterized demodulation and adaptive nonlinear least squares frameworks are employed to enhance signal extraction and robustness against noise (112). DL models, including deep super-resolution networks and the ReViSe framework, improve the resolution and accuracy of rPPG by processing facial landmarks and filtering non-skin areas (66). Additionally, adaptive skin segmentation and multi-wavelength photoplethysmography optimize the region of interest and utilize different wavelengths to enhance measurement accuracy across various skin tones (113, 114).

### 5.2 Environmental challenges

Signal interference and environmental attenuation, particularly in radar-based systems, are exacerbated by obstacles such as walls and furniture, which can attenuate signals and introduce noise (18). The presence of multiple individuals further complicates signal processing, necessitating advanced algorithms to isolate vital signs from background noise. Additionally, low-resolution images can degrade the performance of rPPG systems, which rely on facial video data for HR estimation (115). Techniques like super-resolution processing are crucial for recovering rPPG information and improving measurement accuracy, especially in group settings (115). Advanced algorithms, such as parameterized demodulation and adaptive nonlinear least squares frameworks, enhance the extraction of vital sign information from noisy signals, improving the signal-to-noise ratio and allowing accurate tracking even during small body movements (112).

### 5.3 Privacy and ethical challenges

The integration of AI in non-contact vital sign monitoring technologies significantly enhances the accuracy and efficiency of vital sign acquisition. However, this reliance on AI introduces substantial challenges concerning data privacy, ethics, and security. The sensitive nature of medical data requires robust security measures, especially as 5G networks facilitate faster and more extensive data transfer. Techniques such as encryption, federated learning, and differential privacy are essential to protect patient information and ensure ethical handling of data (116). These measures are crucial to maintaining trust and compliance in the deployment of AI-driven health monitoring systems, addressing both technological and ethical concerns in the healthcare domain.

**FL** offers a decentralized approach to AI training, allowing institutions to improve ML models without sharing raw patient data. This approach allows healthcare providers to collaborate on model refinement while improving data privacy (117, 118).

Figure 13 shows the core difference between traditional centralized ML and FL. In centralized ML, all patient data is collected and transferred from distributed sources to a central server, where model training occurs. This requires direct access to raw vital sign signals, enabling the server to learn global patterns but creating substantial privacy, security, and compliance concerns. Centralized approaches also introduce risks of data leakage, single-point failures, and communication bottlenecks, especially when dealing with sensitive healthcare data. Although effective in terms of model performance, centralized training often conflicts with patient-centric principles and regulatory constraints such as GDPR and HIPAA (117).

**Figure 13:**
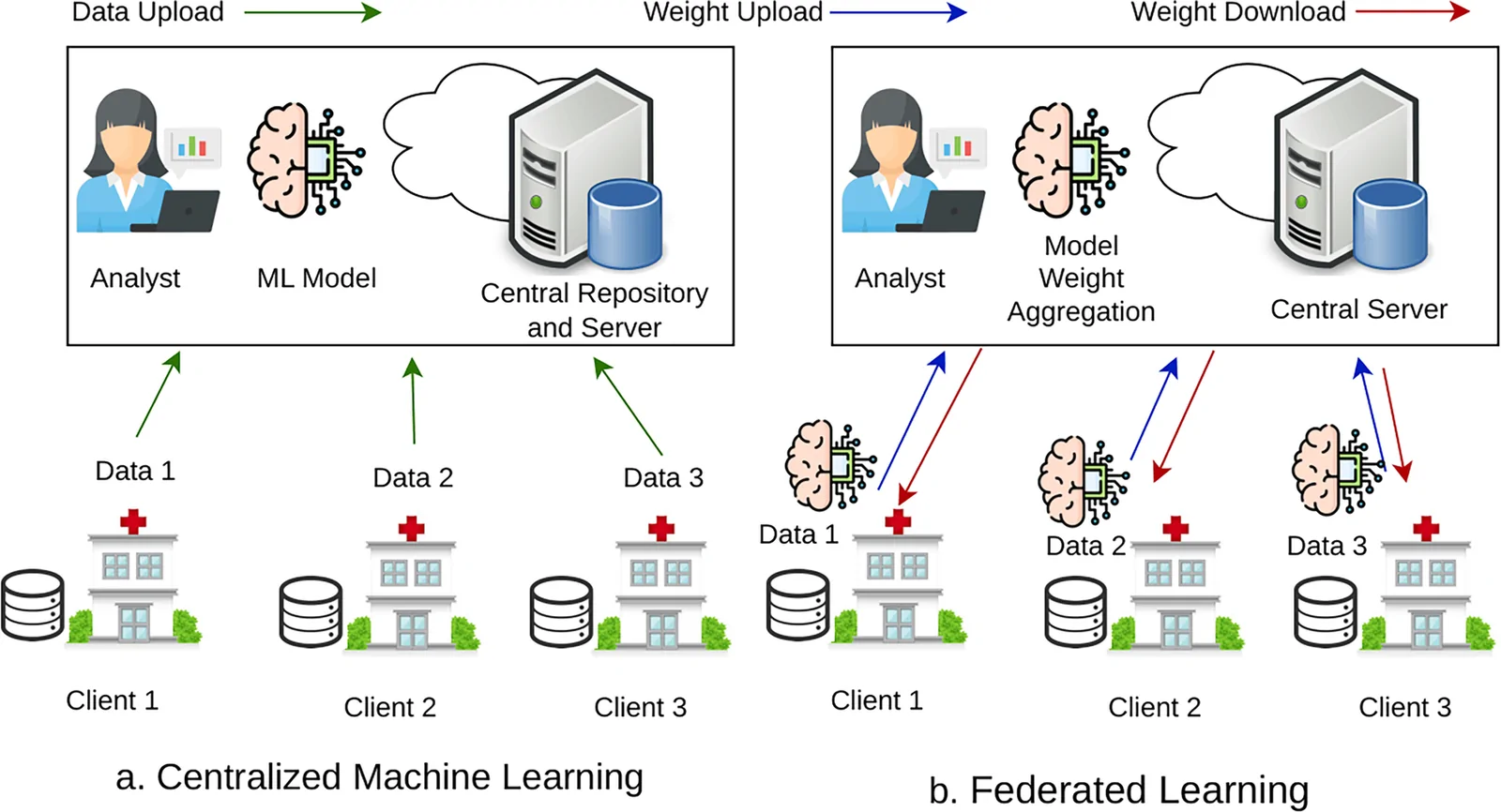
Centralized ML vs FL. In centralized ML **(a)**, raw patient data is uploaded from each client to a central repository where training occurs. In FL **(b)**, training happens locally at each client and only model weights are uploaded for aggregation and downloaded back, so raw data never leaves the institution. (ML: Machine Learning, FL: Federated Learning.)

FL addresses these limitations by enabling model training directly on local devices or institutional silos without transferring raw patient data. Only model updates (such as gradients or parameters) are shared with a central aggregator, preserving data privacy while still benefiting from collaborative learning. This decentralized paradigm reduces communication overhead, enhances security, and supports continuous learning across heterogeneous patient datasets (118, 119). Moreover, modern FL systems incorporate additional security mechanisms such as secure aggregation, differential privacy, homomorphic encryption, and trusted execution environments to further protect sensitive information during communication and model updating (120). These techniques ensure that neither the server nor other clients can infer private details from transmitted updates, strengthening confidentiality and integrity across the learning pipeline. Unlike centralized ML, FL aligns with patient-centric healthcare by keeping sensitive vital sign information securely at the source while still enabling robust predictive modeling (117, 121).

**Blockchain Technology** is another approach that serves as a decentralized, secure, and immutable framework that effectively addresses the privacy and security challenges associated with AI-enhanced non-contact vital sign monitoring systems (122). By leveraging a distributed ledger of transactions, blockchain ensures data integrity, fosters transparency, and builds trust among participants within the healthcare ecosystem. In the context of non-contact vital sign monitoring, blockchain introduces several critical features and benefits. Foremost among these is its ability to provide robust data security, facilitating secure communication and data exchange among monitoring system components through advanced cryptographic protocols (123). Transparency is another defining feature, as blockchain records transactions in real-time, making them fully auditable and fostering accountability among healthcare providers and patients (123). The technology further enables trustless interactions by eliminating the need for intermediaries, allowing healthcare entities to engage directly, simplifying processes, and reducing operational costs (123). Moreover, blockchain’s decentralized architecture enhances system scalability and resilience, supporting the seamless integration of numerous devices and systems within healthcare environments (123). Collectively, these features establish blockchain as an indispensable tool for achieving secure, efficient, and scalable operations in AI-enhanced non-contact vital sign monitoring systems.

## 6 Conceptual framework proposal for vital sign monitoring

This section presents a conceptual framework synthesised from the review findings. It is a design proposal intended to guide future implementation rather than a validated system: no prototype was constructed and no benchmarking was performed. Section 5 outlines several mitigation strategies for addressing the challenges associated with non-contact-based vital sign monitoring systems. Building on these strategies, this section proposes a framework for a non-contact medical IoT system designed for physiological biomarker monitoring, as illustrated in Figure 14. The framework focuses on distributed IoT clients near the patient, each equipped with contactless sensing technologies, including radar, acoustic, vision-based, Wi-Fi, and sensor fusion, to unobtrusively monitor physiological biomarkers without physical contact.

**Figure 14:**
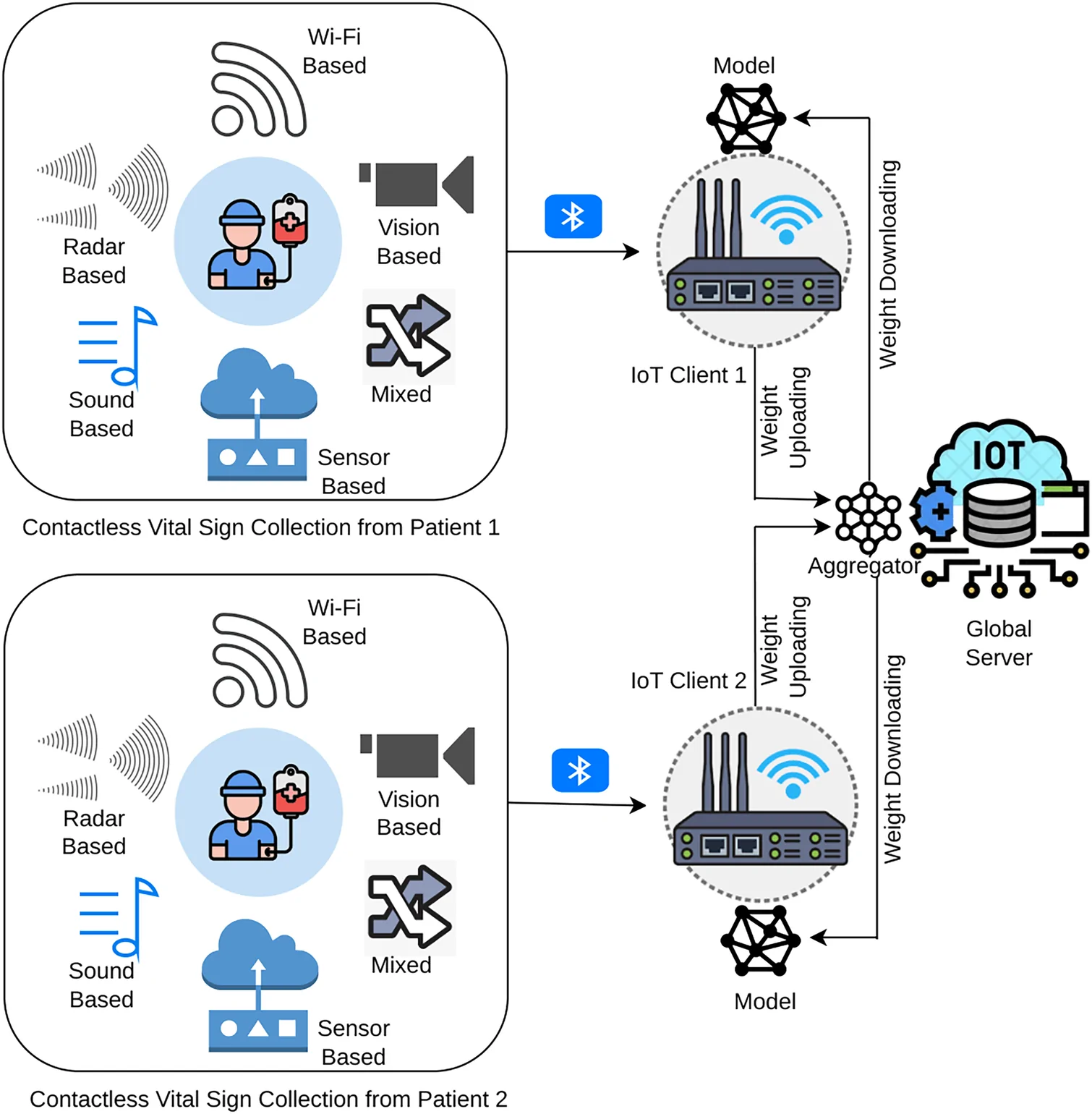
Proposed conceptual framework for non-contact medical IoT physiological biomarker monitoring.

### 6.1 Federated learning formulation

Let K edge clients each hold a local dataset $D_{k}$ of size $n_{k}$, with $n=\sum_{k}n_{k}$. Each client minimises the empirical risk $F_{k}(w)=n_{k}^{-1}\sum_{i\in D_{k}}\ell (w;x_{i},y_{i})$ over its own data, where ℓ(⋅) is the per-sample loss for the estimated vital sign, and the federation minimises the size-weighted global objective $F(w)=\sum_{k}(n_{k}/n)F_{k}(w)$. The aggregator forms the global model at round t+1 as

$$w^{t+1}=\sum_{k=1}^{K}\frac{n_{k}}{n}\;w_{k}^{t}.$$ (21)

Raw physiological data never leaves the client; only the parameter vectors $w_{k}^{t}$ are transmitted, which is the property on which the privacy argument below rests. Since (Equation 21) is size-weighted, clients contributing fewer observations exert proportionally less influence, which matters here because contactless modalities differ substantially in sampling rate and signal quality.

### 6.2 Communication cost and the choice of BLE

For sensor-to-edge communication the relevant quantity is energy per transmitted bit rather than raw throughput, since the payload is a low-rate physiological waveform. A stream sampled at $f_{s}$ with q-bit quantisation across C channels generates $f_{s}qC$ bits per second, which for HR and RR lies orders of magnitude below the capacity of either BLE or Wi-Fi. The choice is therefore governed by per-transmission protocol overhead and idle-state draw rather than bandwidth, both of which favour BLE: its connection-event structure permits the radio to remain dormant for most of the duty cycle, whereas Wi-Fi maintains association overhead irrespective of payload. ZigBee is comparable on energy but lacks native support on consumer clinical devices, which is a deployment rather than an energy constraint.

For the federated link, the per-round client communication cost is

$$C=\beta R_{i}S\left(1+\varepsilon \right),$$ (22)

where $R_{i}$ is the round index, S the model size, ε the serialisation overhead, and β=2 for a fit-only configuration or β=3 when client-side evaluation requires an additional model download. Because ε rises on lower-capability clients, heterogeneous federations incur penalties beyond those predicted by model size alone.

### 6.3 Edge platform selection

The gateway must satisfy three constraints simultaneously: inference must complete within the sampling interval, $T_{\mathrm{pre}}+T_{\inf}+T_{\mathrm{post}}\leq 1/f_{\mathrm{update}}$, where $f_{\mathrm{update}}$ is the required update rate; the model must fit in memory, $M(w)+M_{\mathrm{act}}\leq M_{\mathrm{dev}}$; and per-round energy, given by Equation (23), must remain within the thermal envelope acceptable at the bedside,

$$E_{\mathrm{round}}=P_{\mathrm{idle}}T_{\mathrm{round}}+\Delta P\left(\tau_{\mathrm{train}}+\tau_{\inf}\right)+E_{\mathrm{comm}},$$ (23)

with $\Delta P=P_{\mathrm{active}}-P_{\mathrm{idle}}$ and $E_{\mathrm{comm}}$ following from Equation 22. A compact single-board platform such as the NVIDIA Jetson Orin Nano satisfies these constraints for the update rates relevant to HR and RR while remaining fanless and low-power, which a general-purpose PC does not, and provides throughput a microcontroller-class device lacks. Its affordability enables deployment in resource-limited environments, while also reducing latency, preserving privacy, and allowing offline operation under poor connectivity. Platform-specific values of $P_{\mathrm{idle}}$, $P_{\mathrm{active}}$, and $M_{\mathrm{dev}}$ would need measuring for a given deployment; these constraints define a feasibility region rather than predicting performance.

To maintain signal quality in uncontrolled environments, the framework incorporates edge-side preprocessing techniques to mitigate motion artifacts, skin tone variability, and signal drift. Environmental challenges, such as occlusion, ambient noise, and background clutter, are addressed using super-resolution for low-quality video and radar signal separation in multi-person scenes. These methods, combined with adaptive filtering, enhance system robustness in diverse deployment conditions.

Privacy and ethics were core design principles. The framework adopts FL to train models locally, sharing only model updates, thereby supporting compliance with GDPR and HIPAA. In addition to model-level privacy, the framework recommends FL security mechanisms such as homomorphic encryption to protect sensitive information during communication and aggregation. Homomorphic encryption allows computation over encrypted gradients, preventing the disclosure of raw model updates (120). Under an additively homomorphic scheme with encryption E(⋅), the aggregator evaluates (Equation 21) as $\underset{k}{⨁}E((n_{k}/n)w_{k}^{t})$ without decrypting individual contributions, so no client update is exposed to the server. This comes at the cost of ciphertext expansion, which inflates S in Equation 22 and hence $E_{\mathrm{comm}}$.

FL was selected over blockchain due to the latter’s computational demands, latency, and unsuitability for collaborative learning. While blockchain offers strong auditability, its operational overheads are incompatible with low-power edge deployments. In contrast, FL, designed specifically for distributed AI, offers communication efficiency, scalability, and privacy preservation, making it the preferred choice for this framework.

## 7 Limitations

Our systematic review has certain limitations. Regarding search coverage, eligibility was restricted to peer-reviewed English-language journal articles published between 2019 and 2026, so conference proceedings and preprints were excluded. This restriction is consequential in a field where influential conference venues are established outlets for contact-free sensing research, and it is likely to under-represent Wi-Fi CSI and acoustic sensing work in particular. Its effect is partly mitigated by the inclusion of ACM IMWUT/PACM, which is indexed as a journal while carrying work presented at UbiComp, and which accounts for several of the included studies. In quality assessment, studies were scored independently by two reviewers, with disagreements resolved by consensus; however, only reconciled scores were retained, so inter-rater agreement could not be quantified.

## 8 Conclusion and future scope

This review synthesised 88 peer-reviewed studies on non-contact vital sign monitoring published between 2019 and 2026, covering radar, vision, sound, Wi-Fi, sensor, and mixed system designs. Three findings from the synthesis directly motivate the framework proposed in Section 6. First, the field has consolidated around two sensing paradigms: radar and vision account for 42 and 26 of the 88 studies respectively, while sound, Wi-Fi, sensor, and mixed designs are each represented by fewer than ten, indicating that four of the six modalities remain at an early stage of validation. Second, measurement effort is concentrated on two parameters: RR and HR appear in 83.0% and 67.0% of studies, whereas no other parameter exceeds 11.4%, because RR and HR are recoverable from a dominant periodic component of chest-wall displacement or skin-colour variation, while BP, SpO${}_{2}$, and temperature must be inferred indirectly. Third, quantitative comparison across modalities is constrained by reporting heterogeneity: no vision- or sound-based study in the corpus reports percentage accuracy for HR or RR, and the mixture of accuracy, MAE, RMSE, and correlation across the remainder precludes formal meta-analysis. Alongside these, none of the included studies addressed mitigation strategies for the challenges they identified, and privacy and ethical considerations were largely absent. These gaps shaped the conceptual framework presented in this research. The underrepresentation of privacy-preserving design motivates the federated formulation, in which only parameter vectors leave the client and raw physiological signals remain at source, supporting GDPR and HIPAA compliance; the sensitivity of every modality to uncontrolled conditions motivates edge-side preprocessing for motion artefacts, skin-tone variability, and occlusion; and the low-rate nature of physiological waveforms motivates the energy-per-bit argument for BLE and the latency, memory, and thermal constraints governing edge platform selection. Several directions follow from this scope boundary. The first is to build and test the proposed framework on real hardware, measuring the power and memory values that are treated here only as constraints, and checking how much extra communication cost homomorphic encryption adds. Beyond that, federated clients using different sensing modalities will differ in sampling rate and signal quality, so heterogeneity across clients needs explicit handling rather than simple size-weighted averaging. Combining radar, rPPG, thermal, and depth channels also deserves further study, since no single modality performs well under all conditions.

## Data Availability

The original contributions presented in the study are included in the article/supplementary material, further inquiries can be directed to the corresponding author/s.
