# Supplementary material for: A systematic review of non-contact medical IoT systems for physiological biomarker monitoring
Source: Front Med Technol. 2026 Oct 15;8:1917332. doi: 10.3389/fmedt.2026.1917332 (PMC13716870; doi:10.3389/fmedt.2026.1917332)
Supplement: Supplementary file 1 [file Table1.pdf]

Supplementary Material

1 QUALITY ASSESSMENT OF INCLUDED STUDIES

Table A1: Quality assessment results, with Q1, Q2, etc., corresponding to questions in Table 4 of the main article. Q1–Q4 constitute the methodological rigour subscale and Q5–Q8 the reporting and applicability subscale, each with a maximum of 8. Reference numbers correspond to the reference list of the main article.

Ref.	Validity		Reliability		Applicability				Total	Ref.	Validity		Reliability		Applicability				Total
	Q1	Q2	Q3	Q4	Q5	Q6	Q7	Q8			Q1	Q2	Q3	Q4	Q5	Q6	Q7	Q8	
(84)	2	2	1	1	2	2	2	0	12	(103)	2	2	1	1	2	2	2	0	12
(11)	2	1	1	1	2	2	2	0	11	(104)	2	1	2	1	2	2	2	0	12
(6)	2	2	0	0	2	2	1	0	9	(66)	2	1	0	1	2	2	1	0	9
(9)	2	1	0	1	2	2	2	2	12	(105)	2	1	0	1	2	2	2	1	11
(35)	2	1	0	1	2	2	0	0	8	(106)	2	2	1	0	2	2	2	1	12
(85)	2	2	1	1	2	2	2	0	12	(3)	2	2	1	1	2	2	2	1	13
(45)	2	1	0	0	2	2	2	2	11	(76)	2	2	1	1	2	2	2	1	13
(10)	2	1	1	1	2	2	1	1	11	(75)	1	2	0	1	2	2	2	1	11
(8)	2	2	1	1	2	2	2	0	12	(80)	2	1	0	1	2	2	1	0	9
(16)	2	1	0	0	2	2	2	0	9	(81)	2	2	1	1	2	2	2	1	13
(86)	2	1	0	1	2	2	2	2	12	(44)	2	2	2	1	2	2	2	1	14
(50)	2	2	1	1	2	2	2	1	13	(40)	1	2	2	0	2	2	1	1	11
(87)	2	1	1	1	2	2	2	2	13	(49)	2	1	1	0	2	2	2	0	10
(46)	2	1	0	1	2	2	1	2	11	(42)	2	1	1	0	2	2	1	1	10
(51)	2	1	1	1	2	2	2	0	11	(47)	1	1	2	0	2	2	2	0	10
(88)	2	1	0	1	2	2	2	0	10	(48)	1	1	1	0	2	2	2	0	9
(89)	2	2	1	2	2	2	1	0	12	(43)	1	1	1	0	2	2	2	0	9
(90)	1	0	0	0	2	2	2	1	8	(41)	1	1	2	0	2	2	1	0	9
(91)	2	1	2	1	2	2	2	1	13	(32)	1	1	2	0	2	1	1	0	8
(92)	2	1	1	1	2	2	2	1	12	(37)	1	1	1	0	2	1	1	1	8
(36)	2	1	0	1	2	2	2	1	11	(38)	1	1	1	0	2	2	1	0	8
(93)	2	1	0	1	2	2	2	1	11	(52)	1	1	0	0	2	2	2	0	8
(94)	2	2	1	1	2	2	1	1	12	(33)	1	1	2	0	2	2	1	0	9
(34)	2	1	0	1	2	2	2	1	11	(64)	2	2	2	1	2	2	2	0	13
(39)	2	1	1	0	2	2	2	0	10	(62)	1	2	2	1	2	2	2	1	13
(95)	2	1	1	0	2	2	2	0	10	(54)	2	2	2	0	2	2	2	0	12
(55)	2	1	1	0	2	2	2	0	10	(74)	1	1	2	1	2	2	2	1	12
(96)	2	2	1	1	2	2	2	1	13	(73)	1	1	1	1	2	2	2	1	11
(100)	2	2	1	1	2	2	2	2	14	(71)	2	2	1	0	2	2	2	0	11
(53)	2	1	0	1	2	2	2	0	10	(83)	1	1	1	1	2	2	2	0	10
(56)	2	0	1	1	2	2	2	2	12	(58)	1	1	1	0	2	2	2	1	10
(7)	2	1	0	1	2	2	2	0	10	(82)	1	1	1	1	2	2	1	0	9
(99)	1	0	0	0	2	2	2	2	9	(63)	1	1	1	0	2	2	1	1	9
(60)	2	0	1	0	2	2	2	1	10	(72)	1	1	1	0	2	2	1	1	9
(59)	2	1	0	1	2	2	1	0	9	(61)	1	1	1	0	2	2	1	1	9
(69)	2	1	0	1	2	2	2	2	12	(79)	1	1	1	0	2	2	1	0	8
(70)	2	1	2	1	2	2	2	2	14	(78)	1	1	1	0	2	2	1	0	8
(67)	2	2	1	1	2	2	2	0	12	(57)	1	1	1	1	1	2	1	0	8
(13)	2	2	1	0	2	2	2	0	11	(77)	1	1	2	0	2	2	1	0	9
(101)	2	2	1	1	2	2	2	0	12	(97)	2	2	2	1	2	2	2	1	14
(102)	2	2	1	1	2	2	2	0	12	(107)	2	1	2	2	2	2	1	1	13
(12)	2	1	1	0	2	2	2	0	10	(98)	2	2	2	2	2	2	1	0	13
(65)	2	2	1	1	2	2	2	1	13	(108)	1	1	2	1	2	2	1	1	11
(68)	2	2	1	1	2	2	2	0	12	(109)	1	2	0	1	2	2	2	0	10
